# Molecular Surveillance of EHV-1 Strains Circulating in France during and after the Major 2009 Outbreak in Normandy Involving Respiratory Infection, Neurological Disorder, and Abortion

**DOI:** 10.3390/v11100916

**Published:** 2019-10-04

**Authors:** Gabrielle Sutton, Marie Garvey, Ann Cullinane, Marion Jourdan, Christine Fortier, Peggy Moreau, Marc Foursin, Annick Gryspeerdt, Virginie Maisonnier, Christel Marcillaud-Pitel, Loïc Legrand, Romain Paillot, Stéphane Pronost

**Affiliations:** 1 LABÉO Frank Duncombe, 14280 Saint-Contest, France; christine.fortier@laboratoire-labeo.fr (C.F.); loic.legrand@laboratoire-labeo.fr (L.L.); romain.paillot@laboratoire-labeo.fr (R.P.); stephane.pronost@laboratoire-labeo.fr (S.P.); 2 NORMANDIE UNIV, UNICAEN, BIOTARGEN, 14000 Caen, France; 3 Irish Equine Centre, Johnstown, Naas, County Kildare, Eircode: W91 RH93, Ireland; MGarvey@irishequinecentre.ie (M.G.); ACullinane@irishequinecentre.ie (A.C.);; 4 RESPE, 14280 Saint-Contest, France; technicien@respe.net (M.J.); contact@respe.net (V.M.); c.marcillaud-pitel@respe.net (C.M.-P.);; 5 Clinique équine de la Boisrie, 61500 Chailloué, France; peg_moreau@yahoo.fr (P.M.); clin.eq.laboisrie@laposte.net (M.F.); 6 Equi Focus Point Belgium, 8900 Ypres, Belgium; annick.gryspeerdt@gmail.com

**Keywords:** equine herpesvirus type 1, outbreak, respiratory disease, abortion, neuropathogenic strain, myeloencephalopathy, phylogeny, ORF30, MLST

## Abstract

Equine herpesvirus 1 (EHV-1) is an Alphaherpesvirus infecting not only horses but also other equid and non-equid mammals. It can cause respiratory distress, stillbirth and neonatal death, abortion, and neurological disease. The different forms of disease induced by EHV-1 infection can have dramatic consequences on the equine industry, and thus the virus represents a great challenge for the equine and scientific community. This report describes the progress of a major EHV-1 outbreak that took place in Normandy in 2009, during which the three forms of disease were observed. A collection of EHV-1 strains isolated in France and Belgium from 2012 to 2018 were subsequently genetically analysed in order to characterise EHV-1 strain circulation. The open reading frame 30 (ORF30) non-neuropathogenic associated mutation A_2254_ was the most represented among 148 samples analysed in this study. ORF30 was also sequenced for 14 strains and compared to previously published sequences. Finally, a more global phylogenetic approach was performed based on a recently described Multilocus Sequence Typing (MLST) method. French and Belgian strains were clustered with known strains isolated in United Kingdom and Ireland, with no correlation between the phylogeny and the time of collection or location. This new MLST approach could be a tool to help understand epidemics in stud farms.

## 1. Introduction

Equine herpesvirus 1 (EHV-1) is a member of the *Varicellovirus* genus, in the Alphaherpesvirus sub-family [1]. EHV-1 is known to infect horses as principal hosts but some cases have been reported in other equids and non-equid mammals [2,3,4,5]. Virus transmission occurs through direct contact between horses, infectious aerosols, fomites, and/or indirectly by humans. More recently, transmission of herpesvirus after survival in water was reported. Experimentally, EHV-1 was shown to be stable and infectious in water for over a week under different conditions of pH, salinity, temperature, and turbidity, and up to three weeks under some of these conditions [6]. EHV-1 infection may induce several clinical forms of disease including respiratory infection (also called rhinopneumonitis) associated with pyrexia, cough, and respiratory distress, abortion in pregnant mares, stillbirth and neonatal death, and neurological disorders with symptoms ranging from mild ataxia to complete paralysis of the animal (EHV-1 inducing neurological disease is usually referred as Equine Herpesvirus Encephalomyelitis (EHM)) [7]. However, numerous factors may affect the nature and the extent of clinical signs of disease, such as age, sex, physical condition, prior infection history, and/or the nature of the EHV-1 strain [8]. After infection, the trigeminal ganglia and leukocytes have been identified as the sites of latency for EHV-1 [9,10]. The virus can be reactivated after environmental stimuli (e.g., stress) or therapeutic treatment (e.g., dexamethasone) and replicate in mucous tissues with subsequent dissemination to other hosts [11]. EHV-1 latency mechanisms are poorly understood, although gene regulation has been shown to play a major role in the process [12].

The linear double-stranded genome of EHV-1 is 150 kb long and consists of a unique long (U_L_) and unique short region (U_S_). Each of these regions is surrounded by small inverted sequences (Terminal Repeat Long (TRL)/Internal Repeat Long (IRL) and Terminal Repeat Short (TRS)/Internal repeat Short (IRS), respectively). The genome contains 80 open reading frames (ORFs), some of which show more genetic variability than others and contain sufficient variation for phylogenetic analysis [13]. Previous studies performed ORF and/or whole genome sequencing to study genetic polymorphism [14,15]. Whole genome comparison of two characterised strains, Ab4 [16] and V592 [17], identified a polymorphic region within ORF68 (herpes simplex virus type 1 US2 homologue), which was used to examine the genetic heterogeneity of EHV-1 isolates in several different countries [15,18]. Furthermore, a single point mutation of adenine to guanine at nucleotide position 2254 within ORF30 (DNA polymerase catalytic subunit) is often associated with EHV-1 different forms of disease [19,20,21,22,23,24]. Although it is not exclusive, A_2254_ (non-neuropathogenic) and G_2254_ (neuropathogenic) strains were more frequently associated with abortion and neurological disease, respectively [19,25]. Additionally, genome comparison of Ab4 and V592 identified non-synonymous substitutions that could be used for multi-locus sequence typing (MLST) of EHV-1 strains [19,26].

Due to its impact on animal welfare and performance, EHV-1 represents a major threat to the equine industry and a great interest and challenge for the equine veterinary and scientific community. This report aims to illustrate how challenging EHV-1 infection could be in the field through the description of a multi-syndromic outbreak that lasted over 9 months in 2009 and 2010. This outbreak involved 28 animals and different forms of disease were observed, including two EHM cases and four abortions. Both neuropathogenic and non-neuropathogenic strains were isolated from this major outbreak and were investigated alongside samples collected from 2012 to 2018. Different molecular analytic methods were applied to specimens from these outbreaks (2009, and from 2012 to 2018), including the ORF30 substitution A2254G typing, complete ORF30 sequencing, and Multi Locus Sequence Typing (MLST). These samples included EHV-1 strains isolated from the 2018 EHV-1 epizootic, which is considered to be the most important recorded in France for the last 30 years, with at least 56 outbreaks reported and leading to the cancellation of numerous equestrian events.

## 2. Materials and Methods

### 2.1. EHV-1 Outbreak Data Collection

Data related to EHV-1 outbreaks were collected from the diagnostic and equine research Institute LABEO and the French Epidemiological Surveillance Network for Equine Pathologies (Réseau d’Epidémio-Surveillance en Pathologie Equine (RESPE)) and associated equine veterinary practitioners (see Table 1). From August 2009 to June 2010, 242 samples were collected on a stud farm during one single EHV-1 infectious episode (four tissue samples, 115 nasal swabs, one cerebrospinal fluid, 114 blood samples, and eight vaginal swabs). Concerning this outbreak, which happened on the same premise, mares and foals from the stud farm were divided in six groups while yearlings were kept apart. Mares with foals were divided in groups 1, 2, 3, and 4, while mares without foals were divided in groups 5 and 6. From 2012 to 2018, 180 samples were collected from individual EHV-1 outbreaks by the diagnostic and equine research Institute LABEO (France) (85 tissue samples, 82 nasal swabs, one tracheal liquid, four cerebro-spinal fluids, and 19 blood samples). Detailed information is presented in Supplementary Materials (Table S1).

### 2.2. Biological Samples And Nucleic acids

Samples collected included body fluids (nasopharyngeal swabs, cerebrospinal fluid, tracheal liquid, vaginal swabs), fetal organs (lungs, liver, and placenta), or whole blood samples (EDTA). Nasal swabs were processed in 4 mL Eagle Minimal Essential medium or phosphate-buffered saline (PBS). For nucleic acid extraction, 140 µL of biological fluids extracts, 30 mg of organs, or 2–3 mL of blood were used. DNA was extracted using different extraction kits according to the type of sample: fluid extracts and organs were treated as previously described [27], blood samples were processed using the DNA Blood Maxi Kit (Qiagen, Germany) prior to May 2015, and the NucleoSpin Blood L (Macherey-Nagel, Germany) post May 2015 according to manufacturer’s recommendations. Twenty strains amongst the 180 strains collected were selected for ORF30 sequencing, including three strains collected from outbreaks of EHV-1 in Belgium. Belgian strains were available through a diagnostic collaboration with Equi Focus Point Belgium (equine infectious diseases surveillance network in Belgium) and were incorporated in this study due to the close epidemiological relationship between these two neighbouring countries. Sequenced strains were identified according to their location (French Region or Country) and year of collection, as follows: LOC/ID NUMBER/YEAR.

### 2.3. EHV-1 Identification And Quantification

Real-time Polymerase Chain Reaction (PCR) was performed on purified nucleic acids using Diallo et al., (2006) [28] primers and probe targeting glycoprotein B of EHV-1 (Forward Primer F1: 5′-CAT GTC AAC GCA CTC CCA-3′; Reverse Primer R1: 5′-GGG TCG GGC GTT TCT GT-3′and probe FAM-CCC TAC GCT GCT CC-MGB-NFQ). Viral load quantification was performed as previously described [27,29]. A standard curve based on a cloned sequence was used. Results are expressed as a copy number per mL of nasopharyngeal swab extract. A volume of 2.5 µL of each sample was added to the PCR mixture composed of 12.5 µL TaqMan Universal PCR Master Mix (ThermoFisher Scientific, Waltham, MA, USA), 1.25 µL of each primer (at a concentration of 20 µM) and a defined volume of probe depending on the titration. The mixture was completed with nuclease free water to a final volume of 25 µL.

### 2.4. EHV-1 Strain Typing (ORF30 DNA Polymerase, Position 2254)

Real-time discriminatory allelic PCR was performed on purified nucleic acids using Allen et al. (2007) [22] primers targeting the EHV-1 ORF30 DNA polymerase (Forward Primer 5′-CCA CCC TGG CGC TCG-3′; Reverse Primer 5′-AGC CAG TCG CGC AGC AAG ATG-3′) and probes (“non-neuropathogenic” A2254 Probe VIC-CAT CCG TCA ACT ACT C-MGB; “neuropathogenic” G2254 Probe 6-FAM-TCC GTC GAC TAC TC-MGB). The same mix as described in 2.3. was used for PCR, with 0.6 µL of each primer (at a concentration of 20 µM) and a defined volume of each probe.

### 2.5. ORF30 Sequencing And Analysis

Twenty EHV-1 samples were chosen for ORF30 sequencing, based on the disease type and the year of sampling (see Table S2 in Supplementary Materials). Due to a lesser number of neurological outbreaks and low viral load of these samples of neurological origin, only one sample (BRE/13/2018) isolated during a neurological episode was selected. The EHV-1 strains NORM/1/2009 and NORM/2/2010 correspond to strains isolated from a foal during the 2009 outbreak (Section 3.1.2.) and the strain isolated from the foetus after Mare E abortion (Section 3.1.3), respectively. Biofidal (France) performed primer design and ORF30 (3.665 kb) sequencing. First, a primer set (EHV-1-ORF30-PCR-F: 5′-GAACGTGCGAGTGCTGTTTT-3′ and EHV-1-ORF30-PCR-RC: 5′TGTGAAGGTCTGTTCGACGG-3′) was designed to amplify a 5kb region of EHV-1 including ORF30. The amplification mixture was composed of 5 µL of 5× PrimeSTAR Buffer (Takara, Japan), 2 µL deoxyribo–nucleotide triphosphates (dNTPs) Mix (2.5 mM each), 1 µL of each primer (10 µM), 0.5 µL PrimeSTAR GXL DNA Polymerase (Takara, Kusatu, Shiga, Japan), 13.5 µL RNase Free Water, and 2 µL of sample. Thermocycling conditions were as follows: initial denaturation at 98 °C for 30 s, 35 cycles of denaturation at 96 °C for 20 s, annealing at 61 °C for 20 s, elongation at 68 °C for 5 min, and final elongation at 68 °C for 5 min. Sequencing PCR was subsequently performed using the two external (PCR) primers and nine internal (sequencing) primers detailed in Supplementary Materials (Table S3). Amplicons were purified using ExoSAP-IT ^TM^ kit (ThermoFisher Scientific). Sanger sequencing was performed using Big Dye Terminator Sequencing Mix (ThermoFisher Scientific). Samples were purified before electrophoresis using BigDye XTerminator kit (ThermoFisher Scientific). Then, 3730XL DNA Analyzer was then used for sequence electrophoresis. Single nucleotide polymorphisms (SNP) and consensus sequences were analysed using BioEdit version 7.0.5.3 (Tom Hall, Carlsbad, CA, US) [30] and CodonCode Aligner version 8.0.2 (Codon Code Corporation, Centerville, MA, US) [31] software. Sequences were analysed using MEGA 7 software version 7.2.26 (Koichiro Tamura, Glen Stecher, Sudhir Kumar, the Pennsylvania State University, University Park, PA, US) [32] and phylogenetic trees were built using Neighbour-Joining statistical method [33] and Maximum Composite Likelihood model [34]. Finally, all ORF30 sequences used to build the tree were converted into a Nexus format using Seqret EMBOSS (The European Bioinformatics Institute, Hinxton, Cambridgeshire, UK) [35], and a median joining network was built using Population Analysis with Reticulate Trees (PopART) software (Jessica Leigh, David Bryant and Mike Steel, Dunedin, New Zealand) [36].

### 2.6. Multi-locus Sequence Typing (MLST) Analysis

For Multi Locus Sequence analysis, 37 loci in 26 ORFs were analysed based on non-synonymous changes identified between different published data including Ab4 and V592 protein coding regions as reported by Garvey et al. (2019) [14,19,26]. The MSLT was performed according to methodology described by Garvey et al. (2019) [26]. The method and reactional products used were the same as described by the authors. The MLST based on 37 non-synonymous U_S_ and U_L_ amino acid changes (located in 26 ORFs; ORF2, 5, 8, 11, 13, 14, 15, 22, 29, 30, 31, 32, 33, 34, 36, 37, 39, 40, 42, 45, 46, 50, 52, 57, 73, and 76) was performed on eight EHV-1 strains. A reduced MLST based on 14 of the 37 loci (located in 6 ORFs; ORF11, 13, 30, 37, 52 and 76), suspected to be determinant in clade attribution, was performed on seven other EHV-1 strains (see Supplementary Table S2). Amplification products of target ORFs were purified and sequenced using forward primers (except for ORF13, which needed both forward and reverse sequencing). Sequences were analysed using CodonCode Aligner and BioEdit software. Clade attribution is based on U_L_ sequences distribution and was correlated with MLST segregation by Garvey et al. [14,26]. Sequence analysis was conducted on concatenated amino acids sequences. Both a phylogenetic tree and a network were built using MEGA7 and Splits Tree4 [37], respectively. The phylogenic tree was built using Maximum Likelihood method and Jones Taylor Thornton model, while the network was built using a Neighbour-Net method [38].

### 2.7. Statistical Analysis

Chi-squared was used to test the null hypothesis that there was no correlation between the A_2254_/G_2254_ typing and the form of disease (strains isolated from respiratory cases, abortion cases, or neurological cases).

## 3. Results

### 3.1. The 2009 Multi-Syndromic EHV-1 Outbreak

**Figure 1:**
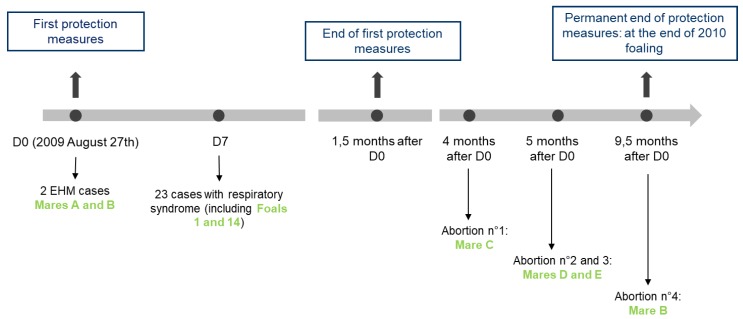
The 2009 EHV-1 outbreak chronology from D0 = day of the first case declaration, to 9.5 months after D0. D7 = Day 7 after the first case was declared. The biosecurity measures that were taken during this outbreak are described in blue boxes.

The outbreak occurred on a thoroughbred stud farm in Normandy that consisted of 169 horses in total (60 broodmares—44 with and 16 without a foal—and 65 yearlings). The first case was reported at the end of August 2009. Two cases of EHM, 23 cases of respiratory disease, and four abortion cases were reported over a period of nine and a half months (see Figure 1). The mares and their foals were kept on one site but separated into six groups (see Table 2). Mares were vaccinated twice a year against equine influenza (EI) with the EIV-HA coding recombinant canarypoxvirus–based vaccine (ProteqFlu-Te, Merial SAS, Lyon, France) and against rhinopneumonitis on the fifth, seventh, and ninth months of gestation with a whole EHV-1/4 inactivated carbomer-adjuvanted vaccine (Duvaxyn EHV1-4, Fort DodgeAnimalHealth, Overland Park, KS, US). Due to reports of EHV-1 outbreaks in other countries in the weeks and months prior to this case (29 August), a booster dose of a whole inactivated EHV-1 vaccine (Pneumequine, Merial) had been given to mares before their return from abroad (Ireland, the UK, and the US), approximately two and a half months before the commencement of the outbreak on the French stud farm. None of the foals had received a primary vaccination in August. Yearlings were kept on a second site located a few kilometers from the main yard. They had received two immunisations one month apart and a booster immunisation six months later (from April 2009 to June 2009, depending on the foals age).

#### 3.1.1. Neurological Cases

• Index Case: Mare A

On 27August 2009 (D0), a pregnant 12-year-old broodmare (information not available concerning pregnancy duration) with an accompanying foal was found in lateral decubitus position and was unable to stand (see Figure 1 and clinical and treatment details are presented in Supplementary Materials S4). Despite treatment and due to a persistent nystagmus, a generalised stiffness and apparition of seizures, the mare was euthanized 14 h after being found in the paddock. Cerebrospinal fluid was collected on the atlantooccipital joint just after the death, and post-mortem examination was performed. EHV-1 was detected by PCR in the cerebrospinal fluid, the brain, and spinal cord. The strain was characterised as “G2254, neuropathogenic” in all three biological compartments.

• Second Case: Mare B

Twelve hours after the onset of clinical signs in mare A, another pregnant mare (11 years old and 3.5 months pregnant, with an accompanying foal from the same group, mare B) presented with depression, shivering, and incoordination from the posterior limbs (see Figure 1 and Supplementary Materials S5). EHV-1 was detected on nasopharyngeal swab and whole blood sample, and typed as “G2254, neuropathogenic.” Forty-eight hours after the first clinical signs were observed, the mare’s condition improved. Mare B made a full recovery one month later.

#### 3.1.2. Respiratory Infections

Between D7 and D10, five foals from group 1 and one foal from group 2 presented with hyperthermia and nasal discharge. Horses were treated depending on their clinical signs. Foals presenting a hyperthermia higher than 39.5 °C received anti-inflammatory drugs (0.3 mg/kg flunixine meglumine intravenously or 17 mg/kg aspirine orally). Foals were also treated with mucolytic drugs (0.5 mg/kg bromhexine orally once a day). Those with a persisting hyperthermia (> 48 h) or more severe clinical signs (coughing, breathing abnormalities) were treated with antibiotics (5 mg/kg trimethoprime-sulfadiazine orally once a day for five days).

Nasopharyngeal swabs were taken from foals and from other animals in each group. Four mares and 13 foals were found positive for EHV-1 in groups 1, 2, and 4, whereas all horses tested in groups 3, 5, and 6 (15 in total) were negative. Further whole blood samples and nasopharyngeal swabs were taken from groups 1, 2, and 4 animals over a six-week period and analysed by qPCR to monitor virus shedding and cell-associated viraemia, respectively. For example, on D7, a foal (foal 1; Table 3) tested positive (high transmission risk) with a high viral load, while foal 14 tested negative. This other foal (foal 14) tested positive on D14, and the viral load significantly increased on D21. It then decreased on D28 and was negative on D42. Overall, virus shedding in foals reached a maximum of 2.14.10^8^ viral particle/mL of nasopharyngeal swab extract between Day 7 and Day 49. Virus loads in blood samples were lower than on swabs extracts and reached a maximum of 2.98.10^3^ viral particle/mL between Day 14 and Day 49 (see Table 3).

#### 3.1.3. Abortion Cases

Between four and five months after the first neurological case (see Figure 1), a mare (C) from group 1 and two mares (D and E) from group 4 aborted and were confirmed EHV-1 positive. Tests were carried out on fetal tissues (liver, lung, and kidney) and the mares’ placentas. Mare C foetus kidney tested negative. The EHV-1 strains were identified as “non-neuropathogenic” (A_2254_) for the three cases.

Six months after developing neurological signs of disease, mare B (9.5 months pregnant) showed signs of dystocic abortion (liquid loss and contractions without delivery of the foal). Intra-uterine examination revealed an anterior dorsal position of the foal and bended legs and head. The foal was moved in the appropriate position under epidural anaesthesia. It was found dead and icteric. Delivery was immediate and complete (see Supplementary Materials S6). The following day, a uterine wash was performed. The mare was isolated in a stable. and sanitary measures were put in place (Section 3.1.4. Biosafety measures). Post-mortem examination of the foetus revealed a densification of lung parenchyma with interlobular oedema. Placenta and lung/liver samples tested positive for EHV-1. The strains were identified as “neuropathogenic” (G_2254_), as previously detected for this mare during this neurological episode (Section 3.1.1.).

Uterine swabs were performed eight days and 30 days post abortion on mares B, C, D and E. All samples were positive for EHV-1 eight days post abortion and negative 30 days post abortion. Mare B was moved to Ireland for breeding five weeks post-abortion. Mare B was confirmed pregnant two months post-abortion and subsequently delivered a healthy foal after a normal pregnancy.

Overall, six of the 60 mares from this stud farm aborted during the year. EHV-1 infection was confirmed for mares B, C, D, and E. One of the two other mares aborted due to *Enterobacter amnigenus* infection, while no cause could be confirmed for the last mare (the dead foal could not be recovered).

#### 3.1.4. Biosafety Measures

The first two cases (Mares A and B) were reported to the RESPE. A safety perimeter was put in place around sick horses. Gloves, gowns, and over-boots were used to manipulate the animals. Movement restrictions were put in place, including foot baths with disinfectant installed at the paddock entrance, car wheels cleaning, and movement restriction for horses.

Mares and foals that tested positive for EHV-1 were moved to isolation from the other horses. All mares were sampled (nasal swabs) before leaving or returning to the stud farm. A negative PCR result for EHV-1 was a prerequisite for movement. Booster vaccination against rhinopneumonitis was also administered. The following year, all pregnant mares stayed in France, including mare B.

To conclude, the overall EHV-1-induced morbidity rate reached 16.6% of the herd (28 clinically affected animals out of 169), including 6.7% of the broodmares, 1.2% EHM cases, and 13.6% respiratory cases.

### 3.2. Surveillance and Phylogeny from 2009 to 2018

#### 3.2.1. Outbreaks, Forms of Disease, and ORF30 A2254G Typing

**Figure 2:**
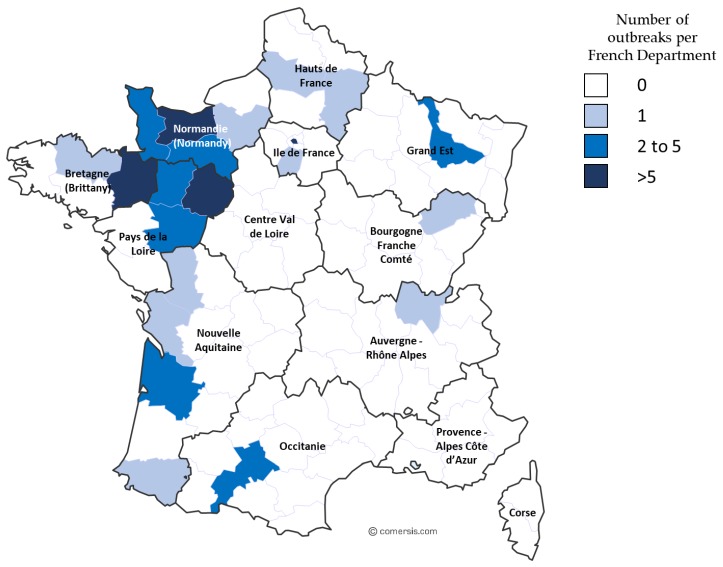
Map of France illustrating EHV-1 outbreaks reported to the RESPE in 2018, with frequency and geographical location.

This epidemiological study involved the 2009 outbreak described in Section 3.1 and EHV-1 outbreaks from 2012 to 2018. Samples collected from 2012 to 2018 are represented in Supplementary Materials (Table S1). From 2012 to 2017, 42 respiratory cases, 44 abortion cases, 20 neurological cases, and six cases with no clinical information were reported. Significantly, in 2018, several outbreaks of EHV-1 occurred. During this year, an increased number of outbreaks (56 in total) were reported to the RESPE, including 15 respiratory outbreaks, 16 cases of abortion, five neurological outbreaks, and 20 outbreaks with no clinical information (see Table 4 and Figure 2). The majority of the outbreaks were reported on the western part of the country, which has the highest concentration of breeding farms. Neurological outbreaks occurred in Normandy and Brittany and lead to the euthanasia of five animals. From these outbreaks, 71 samples were received and analysed (i.e., 15 samples from respiratory cases, 19 samples from abortion cases and 18 samples from neurological cases; clinical information was missing for 19 samples). Of these 71 samples, 12 could not be typed for A2254G substitution (two samples from respiratory cases, one sample from an abortion case, six samples from neurological cases, and three samples missing clinical information).

Among all the strains collected from 2012 to 2018, 137 could be typed for the neuropathogenic/non-neuropathogenic mutation at the ORF30 2254 position. As shown in Table 4, both A_2254_ (non-neuropathogenic type) and G_2254_ (neuropathogenic type) strains have been isolated in respiratory cases, abortion, and neurological outbreaks. The Chi square test indicates a significant statistical association between the ORF30 types and both abortion and neurological disorder (*p*-value = 0.000202, see Table 5). Ninety-one A_2254_ strains have been isolated over the years (66% of all the strains typed), when compared with 46 (34% of all the strains typed) G_2254_ strains_._

#### 3.2.2. ORF30 Sequence Analysis

Probably because of the DNA quantity and purity, only 14 strains could be completely sequenced. BRE/13/2018, the only strain isolated on a neurological outbreak, could not be sequenced. ORF30 sequences from these 14 EHV-1 strains (see Supplementary Table S2) were compared to the neuropathogenic reference strain Ab4 (Genbank accession number AY665713). SNP and amino acid substitutions are reported in Supplementary Table S7.

**Figure 3:**
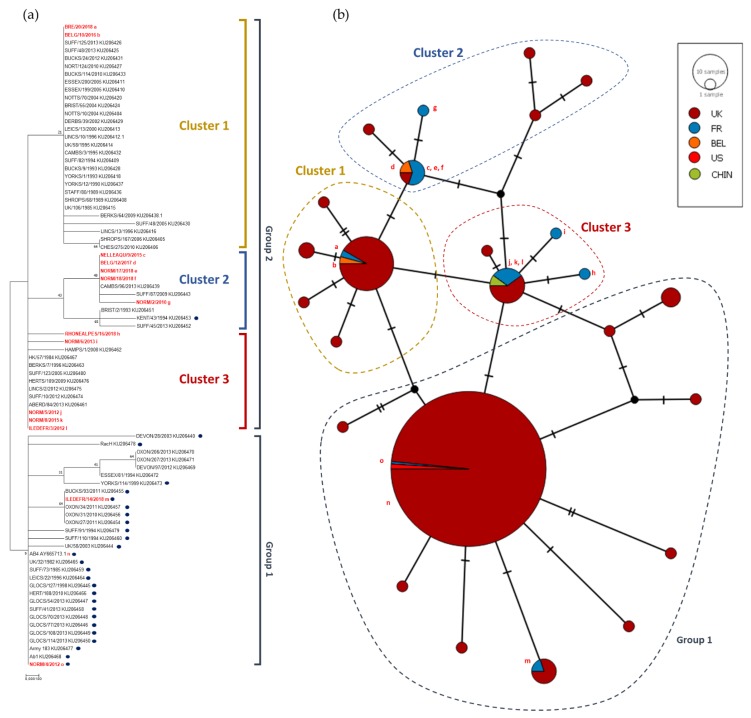
(**a**) Maximum Composite Likelihood method and Tamura-Nei matrix model phylogenetic tree based on ORF30 nucleotide sequences. Eighty-two strains including 14 strains isolated in France and Belgium from 2009 to 2018 (red bold text), 67 strains sequenced by Bryant et al. (2018) [14], and reference strain AY665713 (Ab4) are represented in this tree. Dots represent strains with the neuropathogenic type (G_2254_). Boostrap values after 1000 replication are indicated at major nodes. (**b**) Median Joining Network based on the same ORF30 nucleotide sequences as for the phylogenetic tree.

Twelve of the 14 strains had an adenine residue at position 2254 of the polymerase gene. Seven of these also had a mutation in position 96 (G96A) and five of them in position 2968 (G2968A) compared to reference AY665713 (Ab4). The mutation (A > G) in position 2254 and 2968 led to an amino acid change (N752D and K990E, respectively). Other punctual mutations were observed on the other strains sequenced and five of them induced an amino acid change. One nucleotide on NORM/5/2012 sequence could not be determined during sequencing electrophoresis and was identified as a K (either thymine or guanine). NORM/17/2018 and NORM/18/2018 showed 100% identity at nucleotide and amino acid level. After further investigation about the outbreak location, it appeared that both strains NORM/17/2018 and NORM/18/2018 were collected, respectively, on February 2018 and March 2018 on the same premise after the abortion of two mares present in the stud farm. Although this comparison only concerns ORF30, it is possible that the same strain infected the two pregnant mares and induced their abortion. Both a phylogenetic tree (Neighbour-joining method and Maximum Composite Likelihood model) and a Median Joining Network were constructed (see Figure 3) based on the ORF30 sequences collected since 2009 and sequences obtained in a recent study in UK [14]. Two groups could be observed. The first group (Group 1 in Figure 3) formed a cluster of 32 strains including UK strains and reference strain Ab4. Of these 32, 26 contained the G_2254_ substitution in ORF30 (neuropathogenic type). Both G_2254_ strains collected in France (NORM/4/2012 and ILEDEFR/14/2018) belong to this group. The second group (Group 2 in Figure 3) is divided in three clusters. One of these clusters contains similar strains (NORM/2/2010, NELLEAQU/9/2015, BELG/12/2017, NORM/17/2018, NORM/18/2018) and one UK G_2254_ strain. NORM/5/2012 unidentified nucleotide in position 2876 did not discriminate the strain from ILEDEFR/3/2012 and NORM/8/2015. ORF30 analysis provides a first statement concerning strain phylogeny and potential neuropathogenicity but it only represents a small part of the genome. Multi-locus typing of EHV-1 as described by Garvey et al. 2019 was used to segregate EHV-1 strains into U_L_ clades (Bryant et al. 2018) [14,26].

#### 3.2.3. Phylogeny and Multi-locus Analysis

**Figure 4:**
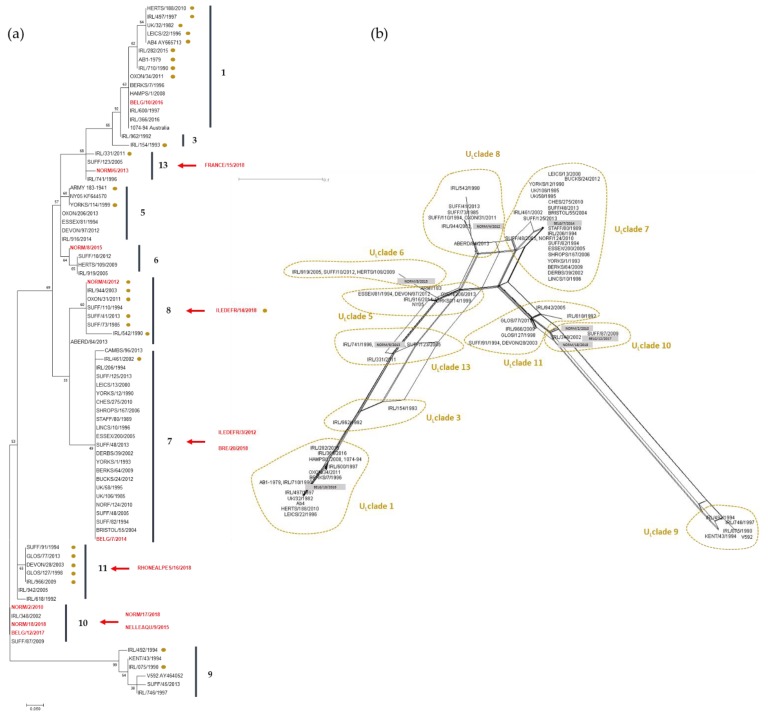
(**a**) Maximum Likelihood phylogenic tree based on Jones Taylor Thornton model built with MLST sequences including 8 French and Belgian strains (in red), 66 EHV-1 UK strains [14], and 22 EHV-1 Irish strains [26]. Dots indicate D752 strains (Neuropathogenic type). Strains with partial concatenated amino acid sequence were not included in the Maximum Likelihood analysis but their supposed position in the tree is indicated with red arrows according to their clade identification (see Table 6). U_L_ Clades [14] are numbered and represented with vertical lines. Boostrap values after 1000 replication are indicated at major nodes. (**b**) Network built using Neighbour-Net method with the same sequences as the tree. Only 37 amino acid sequences were included in the network, and French and Belgian strains are represented in grey boxes.

MLST analysis for the 15 strains is represented in Table 6 and Figure 4. The analysis grouped five strains as U_L_ clade 10 (including two strains isolated from the same outbreak: NORM/17/2018 and NORM/18/2018) and three strains as U_L_ clade 7. Clades 8 and 13 contain two strains each, while clades 1, 6, and 11 contain only one strain. There is no correlation between sites and/or year of collection and clade distribution as also observed with ORF30 sequence comparison. In comparison with ORF30 clusters, strains assigned in group 2 cluster 2 were also assigned in clade 10 according to their MLST profile. The two strains in ORF30 group 1 were assigned to clade 8. No other correlation could be made between ORF30 classification and U_L_ clade classification.

MLST sequences from strains collected in Belgium and France were compared to strains sequenced by Bryant et al. [14], and a Maximum Likelihood phylogenic tree was built based on Jones Taylor Thornton model (see Figure 4).

## 4. Discussion

The 2009 EHV1 outbreak is regretfully a good example of the impact that an EHV-1 infection can have in stud farms and illustrates the diversity of diseases that could be observed and faced by veterinarians. The three clinical forms of disease induced by EHV-1 (respiratory, neurological disorder and abortion) were observed over a nine-and-a-half-month period on a thoroughbred farm (169 horses), which raises questions about the source of EHV-1 infection and its transmission during this outbreak. The significance of this 2009 EHV-1 outbreak was also the observation of the three clinical forms of disease in the same premise over a long period of time. This phenomenon is rarely described in literature [39], and no other outbreak involving all three forms of disease was reported in France since 2009. The number of horses on this premise and the breeding activities may be factors that have contributed to increase the number of cases, which could have been higher in the absence of vaccination. The identification of two different strains, which may be the result from frequent horse movements in France and abroad, might also have influenced the diseases observed during this unusual outbreak. EHV-1 outbreaks are frequent but are usually limited to the report of one or two forms of the disease for the same outbreak (RESPE, personal communication). However, epidemiological links between outbreaks separated in time are not always available or identified. For example, the 2009 outbreak described here lasted nine and a half months with at least two and a half months between the last respiratory infection and the first abortion. Occurrence of multiple forms of disease may be more frequent than currently imagined.

The three clinical forms of disease were reported on a regular basis from 2009 to 2018 on the different outbreaks of this study. In 2018, a large number of cases were reported during a short period (March 2018 to May 2018), with exceptional sanitary measures needed to control contaminations between horses. This EHV-1 crisis is likely to be associated with the fact that a vaccine shortage occurred in 2016, implying a lower vaccination rate.

At the time of the 2009 outbreak, only a few tools were available to conduct EHV-1 molecular investigation. The PCR designed by Diallo et al. (2006, [28]) was used as an EHV-1 detection and quantification test. Viral loads of samples were quantified, and sanitary measures were lifted when undetected. Results obtained at the time indicated that virus titers in total blood samples were lower than in swab extracts, but these two compartments are not always correlated to each other. Monitoring viral loads provided an overview of the virus excretion and risk of transmission. When none of the group 1 foals tested positive after a seven-week isolation period, the day to day management of the stud farm returned to normal. EHV-1 strains were typed (ORF30 SNP A2254G) and both types were identified (neuropathogenic and non-neuropathogenic).

Although those tools gave useful indicative information concerning the different strains isolated from this outbreak, they proved to be limited to establish potential relationship between cases and virus strains. Other tools have been developed (Nugent et al. 2006, Bryant et al. 2018 and Garvey et al. 2019 [14,19,26]) that have motivated our retrospective and molecular analysis of French and Belgian EHV-1 strains isolated from 2009 outbreak to the major 2018 EHV-1 epizootic. Three different molecular analysis were performed: the ORF30 A2254G typing, the complete ORF30 sequencing and the MLST. In 2006, Nugent et al. [19] reported a significant association between the A2254G SNP in the DNA polymerase gene (ORF30), neuropathogenicity. This single point substitution (A2254G) involves an amino acid change (N752D). N752 (A_2254_) strains were strongly associated to non-neuropathogenic infection cases, while D752 (G_2254_) strains were strongly associated to neuropathogenic cases [19]. A2254G typing was subsequently used on a regular basis for strain discrimination [40,41]. In our study, 137 strains collected from 2009 to 2018 were typed, and A_2254_ strains were more significantly associated with abortion cases than to neurological cases (*p* = 0.0002), as recently described by Lechmann et al. (2019) [42]. However, no significant correlation between G_2254_ strains and the neurological form of disease was measured. This observation is in agreement with some recent studies showing that A2254G mutation is not exclusively associated to EHM but could be part of a more complex mechanism affecting strains virulence [41]. Both neuropathogenic and non-neuropathogenic strains could be typed among 2018 strains, suggesting that more than one strain was circulating during the crisis. Strain A2254G typing is also interesting as it was reported that the point mutation could change sensitivity to some drugs targeting DNA polymerase activity as it was demonstrated in a study showing that a N752 variant was more sensitive to aphidicolin than the D752 variants. Aphidicolin inhibits some dNTPs binding to a family of DNA polymerase which include herpesvirus DNA polymerase [17,19].

The A2254G typing was completed with ORF30 sequencing on 14 French and Belgian strains isolated between 2012 and 2018 in order to compare sequences and to perform a phylogenetic analysis. Several synonymous and non-synonymous substitutions were identified. Equine herpesvirus DNA polymerase subunit structures and mechanisms are not well known. Despite a low homology between EHV-1 and Human Simplex Virus (HSV) polymerase amino acid sequence (54%), the latter has been described as closest to α polymerase structure [43]. On this basis, SNP found in EHV-1 strains could be attributed to structure domains identified in HSV polymerase [43]. Amino acid substitutions could be localised in the pre-NH2 terminal domain (R59G), in the 3′-5′ exonuclease domain (S419L and R429K), in the palm domain (A694V and D752N), in the thumb domain (E990K). Although some of the domain activities have been studied for human herpesviruses [43,44], it is hard to predict the impact of the substitutions observed in EHV-1 strains on the protein activity. A strong homology (99.86% to 100%) was measured among those strains with no obvious evolutionary tendency. Results were in agreement with those published by Bryant et al. in 2018 [14]. According to ORF30 sequencing, EHV-1 evolution is not linked to sampling location or year of collection, with the exception of the strains NORM/17/2018 and NORM/18/2018 that were isolated from the same stud farm, a few weeks apart, and have similar ORF30 sequences. All G_2254_ strains were grouped in the same cluster, suggesting that ORF30 sequencing does not provide further information when compared with the A2254G typing. However, three clusters were identified for A2254 strains, primarily differentiated by one SNP (e.g A2968G). The significance of these different clusters is unknown. MLST analysis provides a more global view concerning EHV-1 strains evolution as it takes into account 37 loci in 26 different ORFs. As observed with ORF30 analysis, there is no obvious correlation between year and location of collection, with the exception of NORM/17/2018 and NORM/18/2018, both located in clade 10, which support a co-circulation of EHV-1 strains from different clades as already described by Bryant et al. and Garvey et al. [14,26]. It is interesting to note that four French strains and one Belgium strain are localised in clade 10. The U_L_ Clade 12 described by Bryant et al. (2018) [14], which contains a strain from the UK, is not shown here. U_L_ Clades 2 and 4 were not represented by any of the European strains analysed in this study. It is important to note that the MLST method cannot distinguish U_L_ Clade 2 and 12 from MLST Clade 1 and 10, respectively [26]. As all the strains compared in this study are from Europe, a broader strain selection would be needed to identify a potential geographical effect on clade differentiation. Finally, two abortion strains isolated from the same premise in a one-month interval after two mare abortions had the exact same MLST profile suggesting that the same strain infected the mares. Obviously, EHV-1 strain surveillance is complicated by the fact that EHV-1 can also establish latency in different sites, implying no viral replication as the viral genome maintains an episomal form blocking transcription and translation of its genes (limited transcription with LATs) [9,11]. This implies that strains circulation and outbreaks are potentially dependent on latency and re-activation.

## 5. Conclusions

To conclude, this study allowed applying and comparing three different typing approaches to conduct a phylogenetic analysis over a six years period. A significant association was measured between EHV-1 induced abortion and the DNA polymerase A_2254_ genotype of related strains, while no disease association was observed with the G_2254_ genotype. This result suggests that the commonly used “neuropathogenic/non-neuropathogenic” designation is not always appropriate. The ORF30 and MLST analysis highlight the diversity of EHV-1 strains circulating in the French equine population and the difficulty to link strain evolution, time of collection, and location. However, the MLST offers new possibilities for EHV-1 epidemiology.

## Figures and Tables

**Table 1:** Number of individual outbreaks of equine herpesvirus 1 (EHV-1) reported to Réseau d’Epidémio-Surveillance en Pathologie Equine (RESPE) from 2012 to 2018.

Year	Respiratory	Abortion	Neurological	ND ^1^	Total
2012	MD ^2^	MD	MD	MD	MD
2013	1	1	3	0	5
2014	14	10	3	0	27
2015	11	16	1	0	28
2016	11	8	7	3	32
2017	5	9	6	3	27
2018	15	16	5	20	56

^1^ ND = clinical form of disease not defined or reported. ^2^ MD = missing data.

**Table 2:** Group composition for mares and foals on the French stud farm.

	Mares				Foals		
Group	Total Number	Respiratory Disease	Abortion ^1^	EHM	Total Number	Sex	Respiratory Disease
1	13	0	3 ^1^	2	13 ^2^	Female	6
2	9	0	1	0	9 ^2^	Male	4
3	10	0	0	0	10 ^3^	Female	0
4	12	4	2 ^1^	0	12 ^3^	Male	9
5	6 ^2^	0	0	0	0	0	0
6	10 ^3^	0	0	0	0	0	0

^1^ only four out of six were EHV-1-induced abortion (cf Section 3.1.3.). ^2^ returned from/born abroad. ^3^ stay in/born in France.

**Table 3:** Monitoring of the viral shedding on 14 horses from groups 1, 2, and 3 over a period of seven weeks (copy/mL).

		D7		D14		D21		D28		D35		D42		D49	
	Horse	NS	Blood	NS	Blood	NS	Blood	NS	Blood	NS	Blood	NS	Blood	NS	Blood
**Group 1 and Group 2**	**1**	2.14E + 08	nd	6.75E + 05	1.05E + 03	7.04E + 02	NEG	POS	NEG	NEG	NEG	NEG	NEG	NEG	NEG
	**2**	3.02E + 08	nd	4.52E + 04	NEG	7.04E + 02	NEG	6.05E + 03	NEG	NEG	NEG	NEG	NEG	NEG	NEG
	**3**	9.29E + 07	nd	NEG	NEG	NEG	NEG	8.09E + 02	NEG	2.14E + 03	4.58E + 02	1.11E + 05	NEG	NEG	NEG
	**4**	1.91E + 06	nd	1.81E + 05	2.98E + 03	7.34E + 04	NEG	NEG	POS	NEG	NEG	NEG	NEG	NEG	NEG
	**5**	1.66E + 06	nd	NEG	NEG	3.67E + 04	NEG	NEG	NEG	POS	NEG	NEG	NEG	NEG	NEG
	**6**	1.08E + 07	nd	1.30E + 04	NEG	1.23E + 03	NEG	6.05E + 03	NEG	POS	NEG	NEG	NEG	NEG	NEG
	**7**	2.23E + 05	nd	NEG	POS	POS	NEG	NEG	POS	NEG	POS	NEG	NEG	NEG	NEG
	**8**	1.28E + 05	nd	3.93E + 04	POS	POS	NEG	NEG	NEG	NEG	NEG	NEG	NEG	NEG	NEG
	**9**	4.84E + 04	nd	NEG	NEG	NEG	POS	NEG	NEG	NEG	NEG	NEG	NEG	NEG	NEG
	**10**	NEG	nd	NEG	POS	NEG	NEG	NEG	NEG	NEG	NEG	NEG	NEG	NEG	NEG
	**11**	NEG	nd	NEG	NEG	NEG	NEG	NEG	NEG	NEG	NEG	NEG	NEG	NEG	NEG
	**12**	POS	nd	NEG	NEG	NEG	NEG	NEG	NEG	NEG	NEG	NEG	NEG	NEG	NEG
	**13**	NEG	nd	NEG	NEG	NEG	NEG	NEG	NEG	NEG	5.64E + 02	NEG	NEG	NEG	NEG
	**14**	NEG	nd	6.75E + 05	NEG	1.31E + 08	NEG	POS	NEG	6.48E + 03	NEG	NEG	NEG	NEG	NEG
	**15**	nd	nd	nd	nd	POS	NEG	POS	NEG	NEG	NEG	POS	NEG	NEG	NEG
**Group 4**	**16**	2.26E + 04	nd	NEG	POS	NEG	NEG	NEG	NEG	NEG	NEG	NEG	NEG	NEG	NEG
	**17**	1.18E + 06	nd	4.27E + 03	NEG	NEG	NEG	NEG	NEG	NEG	NEG	NEG	NEG	NEG	NEG
	**18**	1.97E + 04	nd	POS	NEG	NEG	NEG	NEG	NEG	NEG	NEG	NEG	NEG	NEG	NEG

Viral load in nasal swab (NS) from D0 to D42, and in blood from D7 to D42. POS = positive samples non quantifiable; NEG = negative samples. The boxes in gray indicate the period when the horse became negative (when both the NS and blood samples from horses were negative).

**Table 4:** Details of EHV-1 strains, per year, disease type, and strain typing (ORF30 A2254G).

		Respiratory Syndrome		Abortion		Neurological Syndrome		ND ^1^			
Year	Strains	A_2254_	G_2254_	A_2254_	G_2254_	A_2254_	G_2254_	A_2254_	G_2254_	No A/G Typing	Outbreaks (RESPE)
1 major outbreak 2009	30	24	0	2	2	0	2	0	0	0	**/**
2012	8	0	0	0	2	0	1	0	0	5 *	Missing data
2013	19	0	0	6	7	2	1	0	1	2 *	4
2014	10	0	0	8	0	0	0	0	0	2 *	26
2015	32	0	0	17	0	1	1	1	0	12 *	27
2016	18	1	0	8	0	2	4	0	1	2 *	32
2017	22	1	0	2	1	0	7	1	2	8 *	23
2018	71	9	4	17	1	10	2	5	11	12 *	56

Abbreviations: ND ^1^= clinical form of disease not defined or reported. * Respiratory syndrome was assigned only if corresponding clinical information was submitted with nasal swab samples. / = for 2009, all EHV-1 strains included in this study were linked to the outbreak described in Section 3.1.

**Table 5:** ORF30 2254 mutation according to disease from EHV-1 isolates (2009 to 2018).

	Type	Respiratory	Abortion	Neurological	Information Missing	Total
A_2254_	Non-neuro.	11 (73%)	58 (84%)	15 (48%)	7 (32%)	91(66%)
G_2254_	Neuro.	4 (27%)	11 (16%)	16 (52%)	15 (68%)	46 (34%)
		Chi square *p*-value	Resp./Ab.	0.325642		
			Resp./Neu.	0.109608		
			Ab./Neu.	0.000202 *		
			All three	0.000996 *		

Non-neuro. = non neuropathogenic; Neuro. = neuropathogenic. (%: percentage of ORF30 2254 type among the disease category). Chi Square test null hypothesis “There is no correlation between the disease category and the ORF30 2254 type. Resp./Ab. = Chi square test for Respiratory and Abortion categories; Resp./Neu.= Chi square test for Respiratory and Neurological categories; Ab./Neu.= Chi square test for Abortion and Neurological categories; All three= Chi square test for Respiratory, Abortion and Neurological categories. *significant result at *p*-value *p* < 0.05.

**Table 6:** Multi Locus Sequence Typing of 15 EHV-1 strains collected from 2010 to 2018. Determinant loci for clade identification are shaded in grey. Ab4 and V292 are included as reference strains.

ORF	U_L_/MLST Clades	2	5	8	11	11	11	13	13	13	13	13	13	14	14	14	15	22	29	30	30	31	32	33	33	34	36	37	39	40	42	45	46	50	52	57	73	76
LOCI		59	114	114	189	235	250	305	405	460	492	493	499	618/20	628	692	166	430	12	752	990	90	42	15	976	66	47	265	440	196	1275	427	140	367	386	804	122	128
**Ab4 AY665713**	**1**	G	G	D	**Q**	**R**	**A**	**S**	**A**	**A**	**E**	**T**	**A**	---	R	S	D	S	T	**D**	**E**	N	S	N	N	D	S	**A**	S	R	K	E	F	P	**A**	K	A	**F**
**NORM/2/2010**	**10**	D	V	D	**Q**	**R**	**A**	**L**	**A**	**T**	**E**	**T**	**A**	PSR	R	S	N	S	T	**N**	**K**	S	S	H	D	D	S	**V**	S	R	K	G	S	P	**V**	R	A	**S**
**ILEDEFR/3/2012**	**7**				**Q**	**M**	**A**	**S**	**A**	**T**	**E**	**T**	**A**							**N**	**E**							**V**							**V**	R		**S**
**NORM/4/2012**	**8**	D	G	D	**Q**	**R**	**A**	**S**	**A**	**T**	**E**	**T**	**T**	PSR	K	N	N	S	T	**D**	**E**	N	S	H	D	D	S	**V**	S	R	K	G	S	P	**V**	K	A	**S**
**NORM/6/2013**	**13**	D	G	D	**Q**	**R**	**A**	**S**	**A**	**A**	**E**	**T**	**A**	PSR	R	S	D	S	T	**N**	**E**	N	S	N	D	D	S	**V**	S	R	K	G	S	P	**A**	K	A	**S**
**BELG/7/2014**	**7**	D	G	D	**Q**	**M**	**A**	**S**	**A**	**T**	**E**	**T**	**A**	PSR	K	N	N	S	T	**N**	**E**	S	S	H	D	D	S	**V**	S	R	K	G	S	P	**V**	R	A	**S**
**NORM/8/2015**	**6**	D	G	D	**Q**	**R**	S *	**S**	**T**	**A**	**E**	**T**	**A**	PSR	R	S	N	S	T	**N**	**E**	N	S	H	D	D	S	**V**	S	R	K	G	S	P	**V**	K	A	**S**
**NELLEAQU/9/2015**	**10**				**Q**	**R**	**A**	**L**	**A**	**T**	**E**	**T**	**A**							**N**	**K**							**V**							**V**	R		**S**
**BELG/10/2016**	**1**	D	G	D	**Q**	**R**	**A**	**S**	**A**	**A**	**E**	**T**	**A**	---	R	S	D	S	T	**N**	**E**	N	S	N	N	D	S	**A**	S	R	K	E	F	P	**A**	K	A	**F**
**BELG/12/2017**	**10**	D	V	D	**Q**	**R**	**A**	**L**	**A**	**T**	**E**	**T**	**A**	PSR	R	S	N	S	T	**N**	**K**	S	S	H	D	D	S	**V**	S	R	K	G	S	P	**V**	R	A	**S**
**ILEDEFR/14/2018**	**8**				**Q**	**R**	**A**	**S**	**A**	**T**	**E**	**T**	**T**							**D**	**E**							**V**							**V**	K		**S**
**FRANCE/15/2018**	**13**				**Q**	**R**	**A**	**S**	**A**	**A**	**E**	**T**	**A**							**N**	**E**							**V**							**A**	K		**S**
**RHONEALPES/16/2018**	**11**				**Q**	**R**	**A**	**L**	**A**	**T**	**E**	**T**	**A**							**N**	**E**							**V**							**V**	R		**S**
**NORM/17/2018**	**10**				**Q**	**R**	**A**	**L**	**A**	**T**	**E**	**T**	**A**							**N**	**K**							**V**							**V**	R		**S**
**NORM/18/2018**	**10**	D	V	D	**Q**	**R**	**A**	**L**	**A**	**T**	**E**	**T**	**A**	PSR	R	S	N	S	T	**N**	**K**	S	S	H	D	D	S	**V**	S	R	K	G	S	P	**V**	R	A	**S**
**BRE/20/2018**	**7**				**Q**	**M**	**A**	**S**	**A**	**T**	**E**	**T**	**A**							**N**	**E**							**V**							**V**	R		**S**
**V592 AY464052**	**9**	D	V	N	**K**	**R**	**A**	**L**	**A**	**T**	**E**	**T**	**A**	PSR	R	S	N	P	K	**N**	**K**	S	L	H	D	G	R	**V**	L	H	R	G	S	S	**V**	R	V	**S**

* ORF 11(A250S) was used to identify clade 6 strains in this study.

## Supplementary Materials

The following are available online at https://www.mdpi.com/1999-4915/11/10/916/s1, Table S1: Samples type collected from EHV-1 outbreaks from 2012 to 2018, Table S2: Strains identification, location and year of collection. Table S3. ORF30 sequencing Primers. Supplementary Materials S4: Complementary clinical information concerning Mare A. Supplementary Materials S5: Complementary clinical information concerning Mare B. Supplementary Materials S6: Complementary clinical information concerning Mare B abortion. Supplementary Table S7: ORF30 amino acid alignments.
