# Supplementary material for: Molecular Surveillance of EHV-1 Strains Circulating in France during and after the Major 2009 Outbreak in Normandy Involving Respiratory Infection, Neurological Disorder, and Abortion
Source: Viruses. 2019 Oct 4;11(10):916. doi: 10.3390/v11100916 (PMC6832873; doi:10.3390/v11100916)
Supplement: Supplementary file 1 [file viruses-11-00916-s001.zip › Supplementary Materials S1 and S2.pptx]

## Slide 1
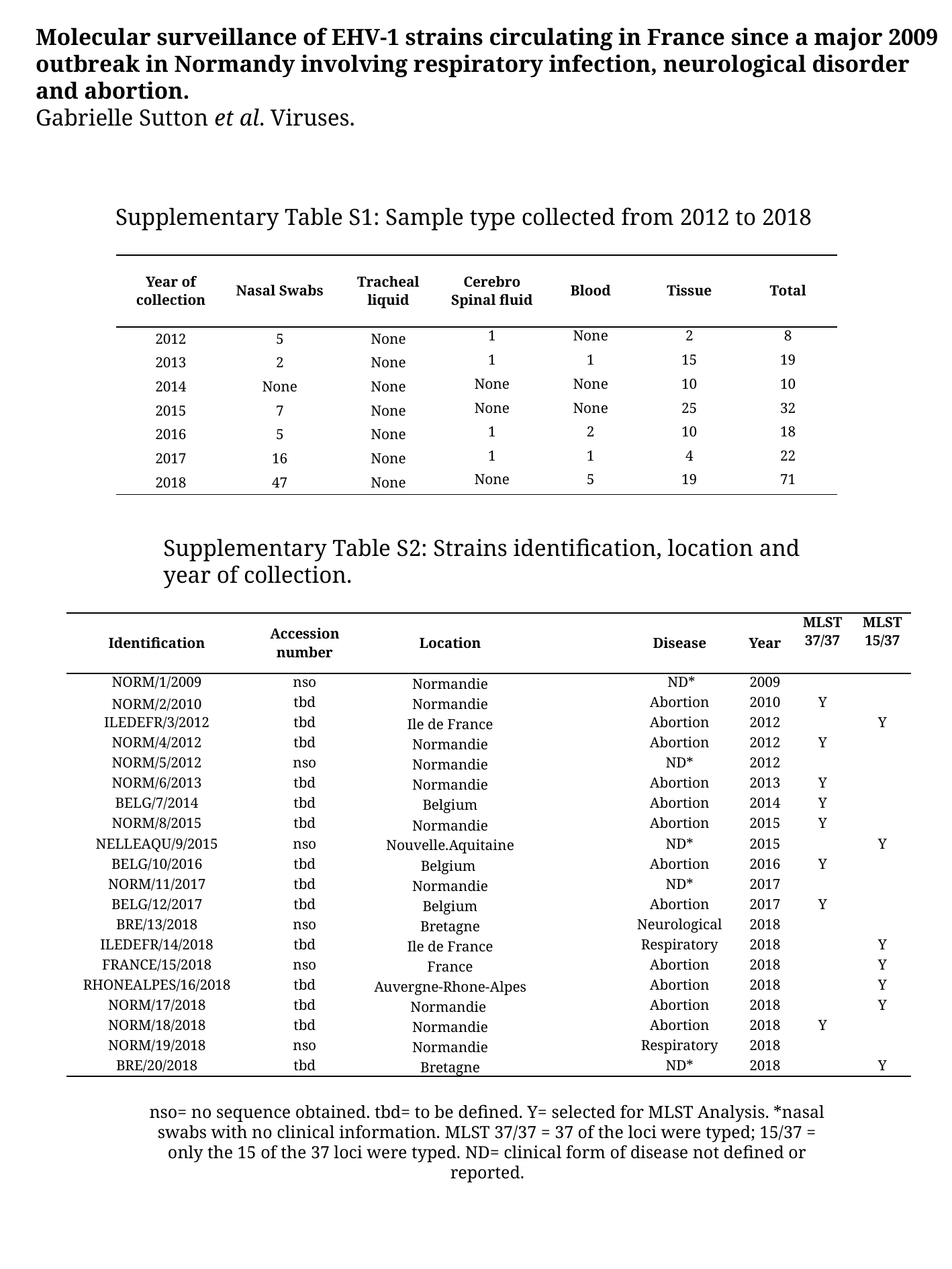

Molecular surveillance of EHV-1 strains circulating in France since a major 2009 outbreak in Normandy involving respiratory infection, neurological disorder and abortion.
Gabrielle Sutton et al. Viruses.
Supplementary Table S1: Sample type collected from 2012 to 2018
| Year of collection | Nasal Swabs | Tracheal liquid | Cerebro Spinal fluid | Blood | Tissue | Total |
| --- | --- | --- | --- | --- | --- | --- |
| 2012 | 5 | None | 1 | None | 2 | 8 |
| 2013 | 2 | None | 1 | 1 | 15 | 19 |
| 2014 | None | None | None | None | 10 | 10 |
| 2015 | 7 | None | None | None | 25 | 32 |
| 2016 | 5 | None | 1 | 2 | 10 | 18 |
| 2017 | 16 | None | 1 | 1 | 4 | 22 |
| 2018 | 47 | None | None | 5 | 19 | 71 |
Supplementary Table S2: Strains identification, location and year of collection.
| Identification | Accession number | Location | | Disease | Year | MLST 37/37 | MLST 15/37 |
| --- | --- | --- | --- | --- | --- | --- | --- |
| NORM/1/2009 | nso | Normandie | | ND\* | 2009 | | |
| NORM/2/2010 | tbd | Normandie | | Abortion | 2010 | Y | |
| ILEDEFR/3/2012 | tbd | Ile de France | | Abortion | 2012 | | Y |
| NORM/4/2012 | tbd | Normandie | | Abortion | 2012 | Y | |
| NORM/5/2012 | nso | Normandie | | ND\* | 2012 | | |
| NORM/6/2013 | tbd | Normandie | | Abortion | 2013 | Y | |
| BELG/7/2014 | tbd | Belgium | | Abortion | 2014 | Y | |
| NORM/8/2015 | tbd | Normandie | | Abortion | 2015 | Y | |
| NELLEAQU/9/2015 | nso | Nouvelle.Aquitaine | | ND\* | 2015 | | Y |
| BELG/10/2016 | tbd | Belgium | | Abortion | 2016 | Y | |
| NORM/11/2017 | tbd | Normandie | | ND\* | 2017 | | |
| BELG/12/2017 | tbd | Belgium | | Abortion | 2017 | Y | |
| BRE/13/2018 | nso | Bretagne | | Neurological | 2018 | | |
| ILEDEFR/14/2018 | tbd | Ile de France | | Respiratory | 2018 | | Y |
| FRANCE/15/2018 | nso | France | | Abortion | 2018 | | Y |
| RHONEALPES/16/2018 | tbd | Auvergne-Rhone-Alpes | | Abortion | 2018 | | Y |
| NORM/17/2018 | tbd | Normandie | | Abortion | 2018 | | Y |
| NORM/18/2018 | tbd | Normandie | | Abortion | 2018 | Y | |
| NORM/19/2018 | nso | Normandie | | Respiratory | 2018 | | |
| BRE/20/2018 | tbd | Bretagne | | ND\* | 2018 | | Y |
nso= no sequence obtained. tbd= to be defined. Y= selected for MLST Analysis. *nasal swabs with no clinical information. MLST 37/37 = 37 of the loci were typed; 15/37 = only the 15 of the 37 loci were typed. ND= clinical form of disease not defined or reported.
