# Supplementary material for: Molecular Surveillance of EHV-1 Strains Circulating in France during and after the Major 2009 Outbreak in Normandy Involving Respiratory Infection, Neurological Disorder, and Abortion
Source: Viruses. 2019 Oct 4;11(10):916. doi: 10.3390/v11100916 (PMC6832873; doi:10.3390/v11100916)
Supplement: Supplementary file 1 [file viruses-11-00916-s001.zip › Supplementary Materials S3.pptx]

## Slide 1
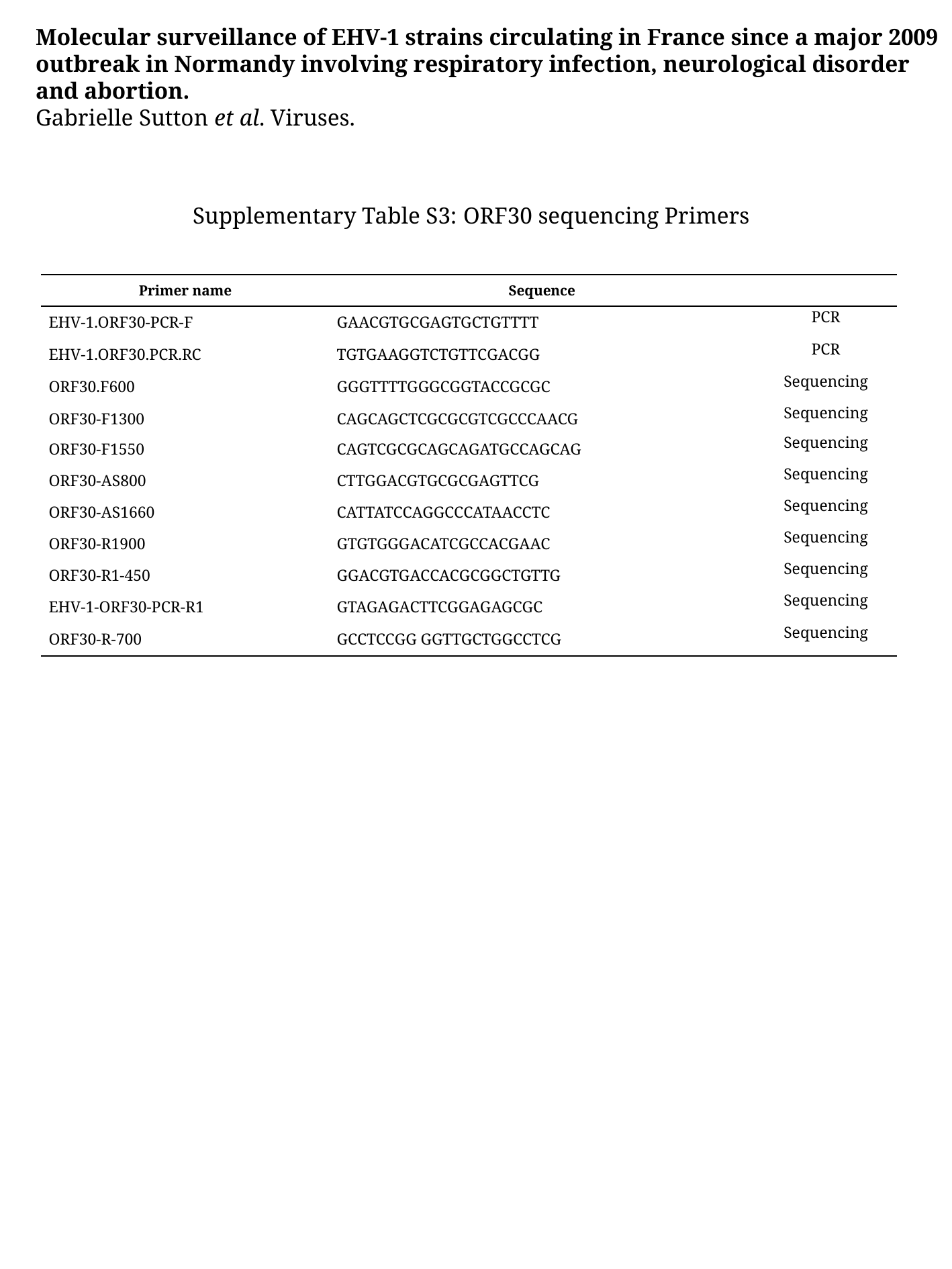

Molecular surveillance of EHV-1 strains circulating in France since a major 2009 outbreak in Normandy involving respiratory infection, neurological disorder and abortion.
Gabrielle Sutton et al. Viruses.
Supplementary Table S3: ORF30 sequencing Primers
| Primer name | Sequence | |
| --- | --- | --- |
| EHV-1.ORF30-PCR-F | GAACGTGCGAGTGCTGTTTT | PCR |
| EHV-1.ORF30.PCR.RC | TGTGAAGGTCTGTTCGACGG | PCR |
| ORF30.F600 | GGGTTTTGGGCGGTACCGCGC | Sequencing |
| ORF30-F1300 | CAGCAGCTCGCGCGTCGCCCAACG | Sequencing |
| ORF30-F1550 | CAGTCGCGCAGCAGATGCCAGCAG | Sequencing |
| ORF30-AS800 | CTTGGACGTGCGCGAGTTCG | Sequencing |
| ORF30-AS1660 | CATTATCCAGGCCCATAACCTC | Sequencing |
| ORF30-R1900 | GTGTGGGACATCGCCACGAAC | Sequencing |
| ORF30-R1-450 | GGACGTGACCACGCGGCTGTTG | Sequencing |
| EHV-1-ORF30-PCR-R1 | GTAGAGACTTCGGAGAGCGC | Sequencing |
| ORF30-R-700 | GCCTCCGG GGTTGCTGGCCTCG | Sequencing |
