# Supplementary material for: Molecular Surveillance of EHV-1 Strains Circulating in France during and after the Major 2009 Outbreak in Normandy Involving Respiratory Infection, Neurological Disorder, and Abortion
Source: Viruses. 2019 Oct 4;11(10):916. doi: 10.3390/v11100916 (PMC6832873; doi:10.3390/v11100916)
Supplement: Supplementary file 1 [file viruses-11-00916-s001.zip › Supplementary Materials S4 to S6.pptx]

## Slide 1
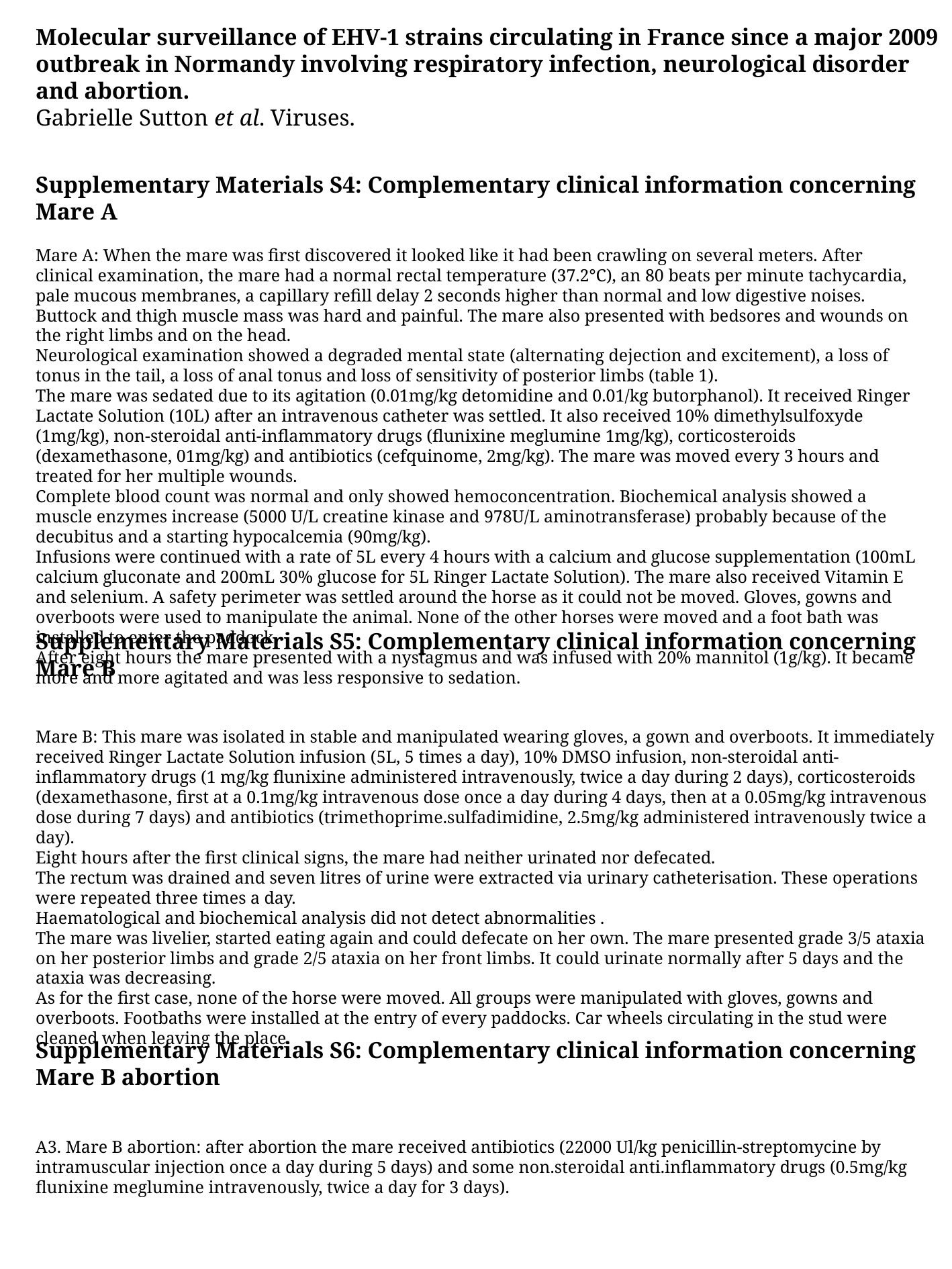

Molecular surveillance of EHV-1 strains circulating in France since a major 2009 outbreak in Normandy involving respiratory infection, neurological disorder and abortion.
Gabrielle Sutton et al. Viruses.
Supplementary Materials S4: Complementary clinical information concerning Mare A
Mare A: When the mare was first discovered it looked like it had been crawling on several meters. After clinical examination, the mare had a normal rectal temperature (37.2°C), an 80 beats per minute tachycardia, pale mucous membranes, a capillary refill delay 2 seconds higher than normal and low digestive noises. Buttock and thigh muscle mass was hard and painful. The mare also presented with bedsores and wounds on the right limbs and on the head.
Neurological examination showed a degraded mental state (alternating dejection and excitement), a loss of tonus in the tail, a loss of anal tonus and loss of sensitivity of posterior limbs (table 1).
The mare was sedated due to its agitation (0.01mg/kg detomidine and 0.01/kg butorphanol). It received Ringer Lactate Solution (10L) after an intravenous catheter was settled. It also received 10% dimethylsulfoxyde (1mg/kg), non-steroidal anti-inflammatory drugs (flunixine meglumine 1mg/kg), corticosteroids (dexamethasone, 01mg/kg) and antibiotics (cefquinome, 2mg/kg). The mare was moved every 3 hours and treated for her multiple wounds.
Complete blood count was normal and only showed hemoconcentration. Biochemical analysis showed a muscle enzymes increase (5000 U/L creatine kinase and 978U/L aminotransferase) probably because of the decubitus and a starting hypocalcemia (90mg/kg).
Infusions were continued with a rate of 5L every 4 hours with a calcium and glucose supplementation (100mL calcium gluconate and 200mL 30% glucose for 5L Ringer Lactate Solution). The mare also received Vitamin E and selenium. A safety perimeter was settled around the horse as it could not be moved. Gloves, gowns and overboots were used to manipulate the animal. None of the other horses were moved and a foot bath was installed to enter the paddock.
After eight hours the mare presented with a nystagmus and was infused with 20% mannitol (1g/kg). It became more and more agitated and was less responsive to sedation.
Supplementary Materials S5: Complementary clinical information concerning Mare B
Mare B: This mare was isolated in stable and manipulated wearing gloves, a gown and overboots. It immediately received Ringer Lactate Solution infusion (5L, 5 times a day), 10% DMSO infusion, non-steroidal anti-inflammatory drugs (1 mg/kg flunixine administered intravenously, twice a day during 2 days), corticosteroids (dexamethasone, first at a 0.1mg/kg intravenous dose once a day during 4 days, then at a 0.05mg/kg intravenous dose during 7 days) and antibiotics (trimethoprime.sulfadimidine, 2.5mg/kg administered intravenously twice a day).
Eight hours after the first clinical signs, the mare had neither urinated nor defecated.
The rectum was drained and seven litres of urine were extracted via urinary catheterisation. These operations were repeated three times a day.
Haematological and biochemical analysis did not detect abnormalities .
The mare was livelier, started eating again and could defecate on her own. The mare presented grade 3/5 ataxia on her posterior limbs and grade 2/5 ataxia on her front limbs. It could urinate normally after 5 days and the ataxia was decreasing.
As for the first case, none of the horse were moved. All groups were manipulated with gloves, gowns and overboots. Footbaths were installed at the entry of every paddocks. Car wheels circulating in the stud were cleaned when leaving the place.
Supplementary Materials S6: Complementary clinical information concerning Mare B abortion
A3. Mare B abortion: after abortion the mare received antibiotics (22000 Ul/kg penicillin-streptomycine by intramuscular injection once a day during 5 days) and some non.steroidal anti.inflammatory drugs (0.5mg/kg flunixine meglumine intravenously, twice a day for 3 days).
