# Supplementary material for: Molecular Surveillance of EHV-1 Strains Circulating in France during and after the Major 2009 Outbreak in Normandy Involving Respiratory Infection, Neurological Disorder, and Abortion
Source: Viruses. 2019 Oct 4;11(10):916. doi: 10.3390/v11100916 (PMC6832873; doi:10.3390/v11100916)
Supplement: Supplementary file 1 [file viruses-11-00916-s001.zip › Supplementary Materials S7bis.pptx]

## Slide 1
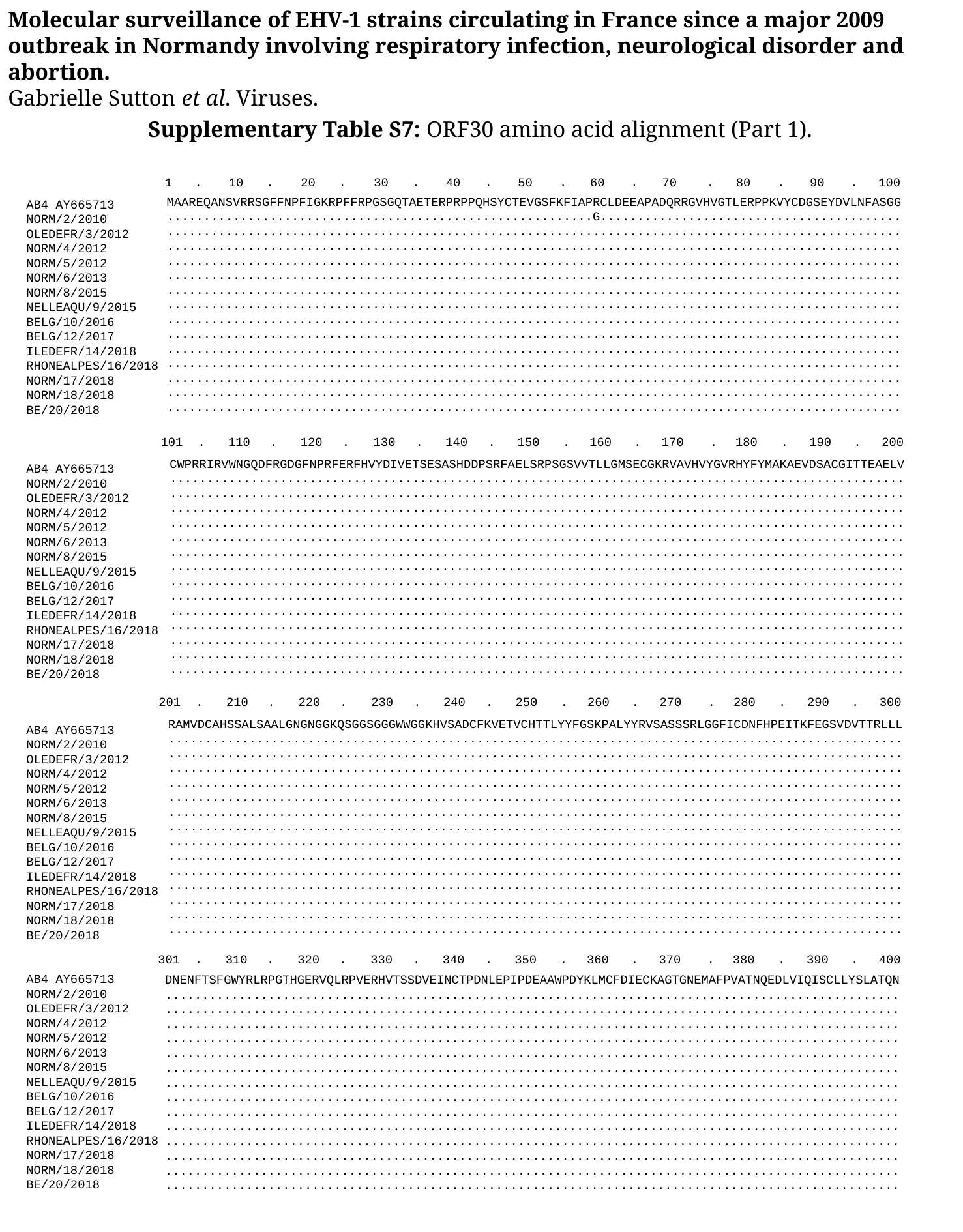

Molecular surveillance of EHV-1 strains circulating in France since a major 2009 outbreak in Normandy involving respiratory infection, neurological disorder and abortion.
Gabrielle Sutton et al. Viruses.
Supplementary Table S7: ORF30 amino acid alignment (Part 1).
1
.
10
.
20
.
30
.
40
.
50
.
60
.
70
.
80
.
90
.
100
MAAREQANSVRRSGFFNPFIGKRPFFRPGSGQTAETERPRPPQHSYCTEVGSFKFIAPRCLDEEAPADQRRGVHVGTLERPPKVYCDGSEYDVLNFASGG
..........................................................G.........................................
....................................................................................................
....................................................................................................
....................................................................................................
....................................................................................................
....................................................................................................
....................................................................................................
....................................................................................................
....................................................................................................
....................................................................................................
....................................................................................................
....................................................................................................
....................................................................................................
....................................................................................................
AB4 AY665713
NORM/2/2010
OLEDEFR/3/2012
NORM/4/2012
NORM/5/2012
NORM/6/2013
NORM/8/2015
NELLEAQU/9/2015
BELG/10/2016
BELG/12/2017
ILEDEFR/14/2018
RHONEALPES/16/2018
NORM/17/2018
NORM/18/2018
BE/20/2018
101
.
110
.
120
.
130
.
140
.
150
.
160
.
170
.
180
.
190
.
200
CWPRRIRVWNGQDFRGDGFNPRFERFHVYDIVETSESASHDDPSRFAELSRPSGSVVTLLGMSECGKRVAVHVYGVRHYFYMAKAEVDSACGITTEAELV
....................................................................................................
....................................................................................................
....................................................................................................
....................................................................................................
....................................................................................................
....................................................................................................
....................................................................................................
....................................................................................................
....................................................................................................
....................................................................................................
....................................................................................................
....................................................................................................
....................................................................................................
....................................................................................................
AB4 AY665713
NORM/2/2010
OLEDEFR/3/2012
NORM/4/2012
NORM/5/2012
NORM/6/2013
NORM/8/2015
NELLEAQU/9/2015
BELG/10/2016
BELG/12/2017
ILEDEFR/14/2018
RHONEALPES/16/2018
NORM/17/2018
NORM/18/2018
BE/20/2018
201
.
210
.
220
.
230
.
240
.
250
.
260
.
270
.
280
.
290
.
300
RAMVDCAHSSALSAALGNGNGGKQSGGSGGGWWGGKHVSADCFKVETVCHTTLYYFGSKPALYYRVSASSSRLGGFICDNFHPEITKFEGSVDVTTRLLL
....................................................................................................
....................................................................................................
....................................................................................................
....................................................................................................
....................................................................................................
....................................................................................................
....................................................................................................
....................................................................................................
....................................................................................................
....................................................................................................
....................................................................................................
....................................................................................................
....................................................................................................
....................................................................................................
AB4 AY665713
NORM/2/2010
OLEDEFR/3/2012
NORM/4/2012
NORM/5/2012
NORM/6/2013
NORM/8/2015
NELLEAQU/9/2015
BELG/10/2016
BELG/12/2017
ILEDEFR/14/2018
RHONEALPES/16/2018
NORM/17/2018
NORM/18/2018
BE/20/2018
301
.
310
.
320
.
330
.
340
.
350
.
360
.
370
.
380
.
390
.
400
AB4 AY665713
NORM/2/2010
OLEDEFR/3/2012
NORM/4/2012
NORM/5/2012
NORM/6/2013
NORM/8/2015
NELLEAQU/9/2015
BELG/10/2016
BELG/12/2017
ILEDEFR/14/2018
RHONEALPES/16/2018
NORM/17/2018
NORM/18/2018
BE/20/2018
DNENFTSFGWYRLRPGTHGERVQLRPVERHVTSSDVEINCTPDNLEPIPDEAAWPDYKLMCFDIECKAGTGNEMAFPVATNQEDLVIQISCLLYSLATQN
....................................................................................................
....................................................................................................
....................................................................................................
....................................................................................................
....................................................................................................
....................................................................................................
....................................................................................................
....................................................................................................
....................................................................................................
....................................................................................................
....................................................................................................
....................................................................................................
....................................................................................................
....................................................................................................

## Slide 2
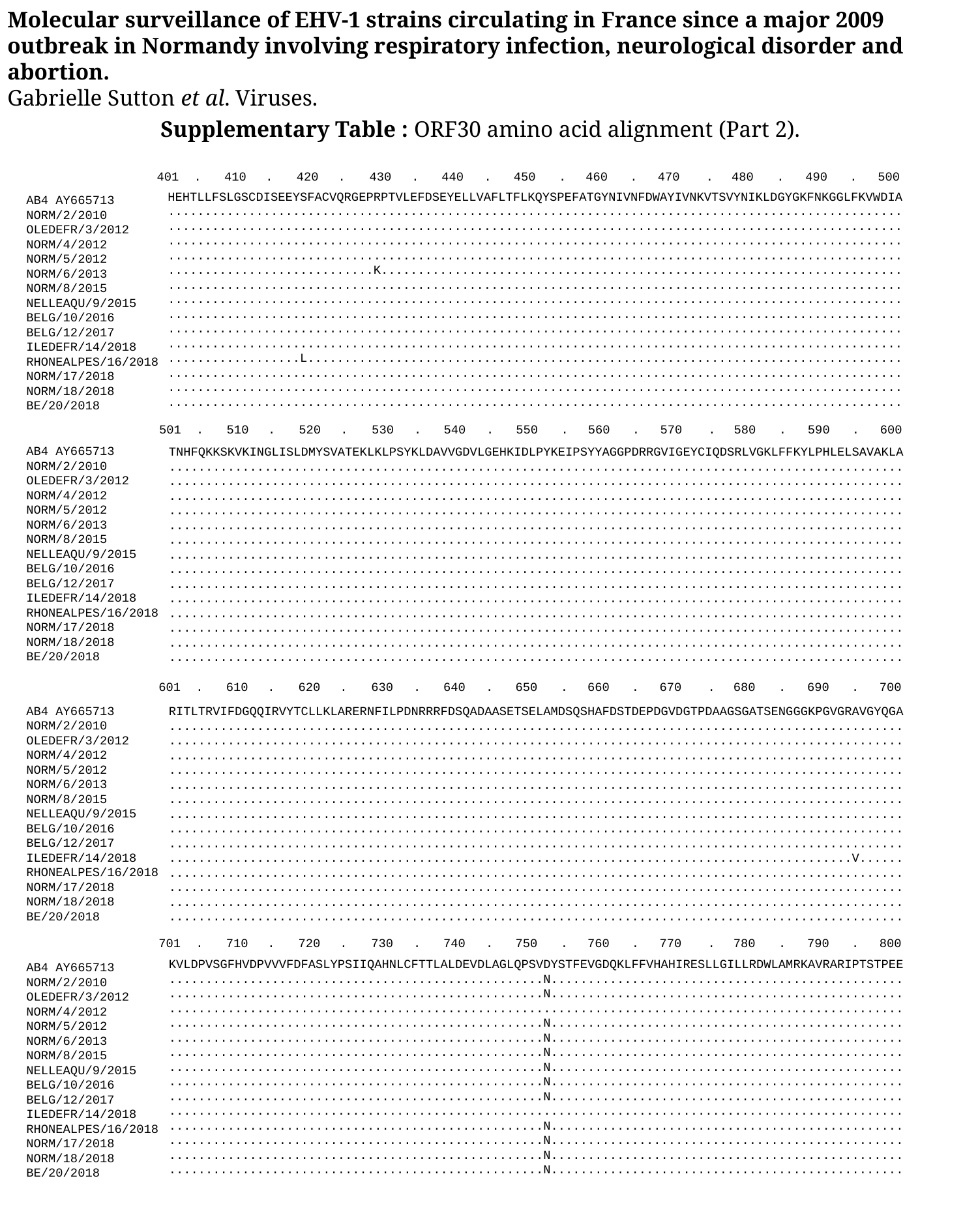

Molecular surveillance of EHV-1 strains circulating in France since a major 2009 outbreak in Normandy involving respiratory infection, neurological disorder and abortion.
Gabrielle Sutton et al. Viruses.
Supplementary Table : ORF30 amino acid alignment (Part 2).
401
.
410
.
420
.
430
.
440
.
450
.
460
.
470
.
480
.
490
.
500
HEHTLLFSLGSCDISEEYSFACVQRGEPRPTVLEFDSEYELLVAFLTFLKQYSPEFATGYNIVNFDWAYIVNKVTSVYNIKLDGYGKFNKGGLFKVWDIA
....................................................................................................
....................................................................................................
....................................................................................................
....................................................................................................
............................K.......................................................................
....................................................................................................
....................................................................................................
....................................................................................................
....................................................................................................
....................................................................................................
..................L.................................................................................
....................................................................................................
....................................................................................................
....................................................................................................
AB4 AY665713
NORM/2/2010
OLEDEFR/3/2012
NORM/4/2012
NORM/5/2012
NORM/6/2013
NORM/8/2015
NELLEAQU/9/2015
BELG/10/2016
BELG/12/2017
ILEDEFR/14/2018
RHONEALPES/16/2018
NORM/17/2018
NORM/18/2018
BE/20/2018
501
.
510
.
520
.
530
.
540
.
550
.
560
.
570
.
580
.
590
.
600
AB4 AY665713
NORM/2/2010
OLEDEFR/3/2012
NORM/4/2012
NORM/5/2012
NORM/6/2013
NORM/8/2015
NELLEAQU/9/2015
BELG/10/2016
BELG/12/2017
ILEDEFR/14/2018
RHONEALPES/16/2018
NORM/17/2018
NORM/18/2018
BE/20/2018
TNHFQKKSKVKINGLISLDMYSVATEKLKLPSYKLDAVVGDVLGEHKIDLPYKEIPSYYAGGPDRRGVIGEYCIQDSRLVGKLFFKYLPHLELSAVAKLA
....................................................................................................
....................................................................................................
....................................................................................................
....................................................................................................
....................................................................................................
....................................................................................................
....................................................................................................
....................................................................................................
....................................................................................................
....................................................................................................
....................................................................................................
....................................................................................................
....................................................................................................
....................................................................................................
601
.
610
.
620
.
630
.
640
.
650
.
660
.
670
.
680
.
690
.
700
AB4 AY665713
NORM/2/2010
OLEDEFR/3/2012
NORM/4/2012
NORM/5/2012
NORM/6/2013
NORM/8/2015
NELLEAQU/9/2015
BELG/10/2016
BELG/12/2017
ILEDEFR/14/2018
RHONEALPES/16/2018
NORM/17/2018
NORM/18/2018
BE/20/2018
RITLTRVIFDGQQIRVYTCLLKLARERNFILPDNRRRFDSQADAASETSELAMDSQSHAFDSTDEPDGVDGTPDAAGSGATSENGGGKPGVGRAVGYQGA
....................................................................................................
....................................................................................................
....................................................................................................
....................................................................................................
....................................................................................................
....................................................................................................
....................................................................................................
....................................................................................................
....................................................................................................
.............................................................................................V......
....................................................................................................
....................................................................................................
....................................................................................................
....................................................................................................
701
.
710
.
720
.
730
.
740
.
750
.
760
.
770
.
780
.
790
.
800
KVLDPVSGFHVDPVVVFDFASLYPSIIQAHNLCFTTLALDEVDLAGLQPSVDYSTFEVGDQKLFFVHAHIRESLLGILLRDWLAMRKAVRARIPTSTPEE
...................................................N................................................
...................................................N................................................
....................................................................................................
...................................................N................................................
...................................................N................................................
...................................................N................................................
...................................................N................................................
...................................................N................................................
...................................................N................................................
....................................................................................................
...................................................N................................................
...................................................N................................................
...................................................N................................................
...................................................N................................................
AB4 AY665713
NORM/2/2010
OLEDEFR/3/2012
NORM/4/2012
NORM/5/2012
NORM/6/2013
NORM/8/2015
NELLEAQU/9/2015
BELG/10/2016
BELG/12/2017
ILEDEFR/14/2018
RHONEALPES/16/2018
NORM/17/2018
NORM/18/2018
BE/20/2018

## Slide 3
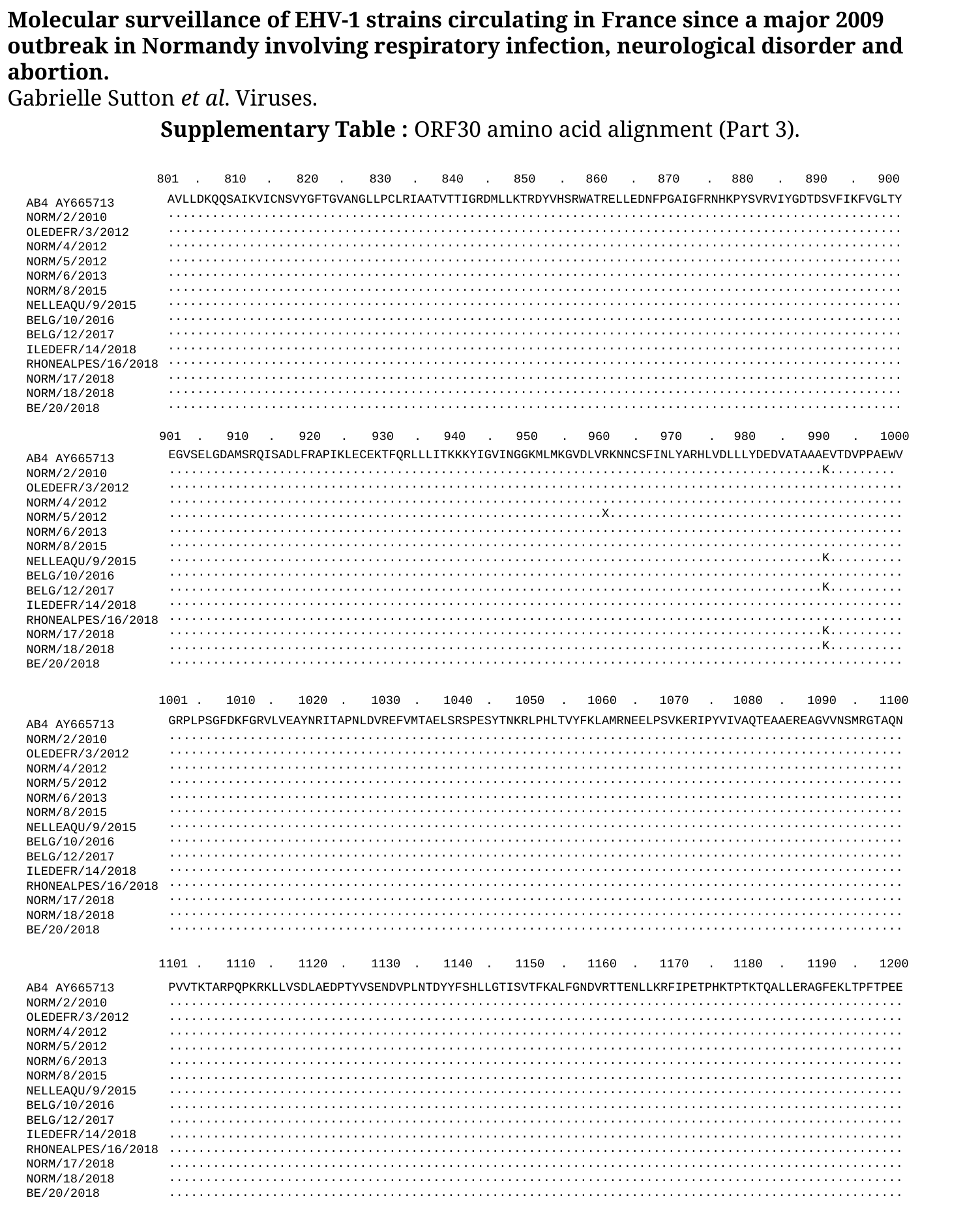

Molecular surveillance of EHV-1 strains circulating in France since a major 2009 outbreak in Normandy involving respiratory infection, neurological disorder and abortion.
Gabrielle Sutton et al. Viruses.
Supplementary Table : ORF30 amino acid alignment (Part 3).
801
.
810
.
820
.
830
.
840
.
850
.
860
.
870
.
880
.
890
.
900
AVLLDKQQSAIKVICNSVYGFTGVANGLLPCLRIAATVTTIGRDMLLKTRDYVHSRWATRELLEDNFPGAIGFRNHKPYSVRVIYGDTDSVFIKFVGLTY
....................................................................................................
....................................................................................................
....................................................................................................
....................................................................................................
....................................................................................................
....................................................................................................
....................................................................................................
....................................................................................................
....................................................................................................
....................................................................................................
....................................................................................................
....................................................................................................
....................................................................................................
....................................................................................................
AB4 AY665713
NORM/2/2010
OLEDEFR/3/2012
NORM/4/2012
NORM/5/2012
NORM/6/2013
NORM/8/2015
NELLEAQU/9/2015
BELG/10/2016
BELG/12/2017
ILEDEFR/14/2018
RHONEALPES/16/2018
NORM/17/2018
NORM/18/2018
BE/20/2018
901
.
910
.
920
.
930
.
940
.
950
.
960
.
970
.
980
.
990
.
1000
EGVSELGDAMSRQISADLFRAPIKLECEKTFQRLLLITKKKYIGVINGGKMLMKGVDLVRKNNCSFINLYARHLVDLLLYDEDVATAAAEVTDVPPAEWV
.........................................................................................K.........
....................................................................................................
....................................................................................................
...........................................................X........................................
....................................................................................................
....................................................................................................
.........................................................................................K..........
....................................................................................................
.........................................................................................K..........
....................................................................................................
....................................................................................................
.........................................................................................K..........
.........................................................................................K..........
....................................................................................................
AB4 AY665713
NORM/2/2010
OLEDEFR/3/2012
NORM/4/2012
NORM/5/2012
NORM/6/2013
NORM/8/2015
NELLEAQU/9/2015
BELG/10/2016
BELG/12/2017
ILEDEFR/14/2018
RHONEALPES/16/2018
NORM/17/2018
NORM/18/2018
BE/20/2018
1001
.
1010
.
1020
.
1030
.
1040
.
1050
.
1060
.
1070
.
1080
.
1090
.
1100
GRPLPSGFDKFGRVLVEAYNRITAPNLDVREFVMTAELSRSPESYTNKRLPHLTVYFKLAMRNEELPSVKERIPYVIVAQTEAAEREAGVVNSMRGTAQN
....................................................................................................
....................................................................................................
....................................................................................................
....................................................................................................
....................................................................................................
....................................................................................................
....................................................................................................
....................................................................................................
....................................................................................................
....................................................................................................
....................................................................................................
....................................................................................................
....................................................................................................
....................................................................................................
AB4 AY665713
NORM/2/2010
OLEDEFR/3/2012
NORM/4/2012
NORM/5/2012
NORM/6/2013
NORM/8/2015
NELLEAQU/9/2015
BELG/10/2016
BELG/12/2017
ILEDEFR/14/2018
RHONEALPES/16/2018
NORM/17/2018
NORM/18/2018
BE/20/2018
1101
.
1110
.
1120
.
1130
.
1140
.
1150
.
1160
.
1170
.
1180
.
1190
.
1200
PVVTKTARPQPKRKLLVSDLAEDPTYVSENDVPLNTDYYFSHLLGTISVTFKALFGNDVRTTENLLKRFIPETPHKTPTKTQALLERAGFEKLTPFTPEE
....................................................................................................
....................................................................................................
....................................................................................................
....................................................................................................
....................................................................................................
....................................................................................................
....................................................................................................
....................................................................................................
....................................................................................................
....................................................................................................
....................................................................................................
....................................................................................................
....................................................................................................
....................................................................................................
AB4 AY665713
NORM/2/2010
OLEDEFR/3/2012
NORM/4/2012
NORM/5/2012
NORM/6/2013
NORM/8/2015
NELLEAQU/9/2015
BELG/10/2016
BELG/12/2017
ILEDEFR/14/2018
RHONEALPES/16/2018
NORM/17/2018
NORM/18/2018
BE/20/2018

## Slide 4
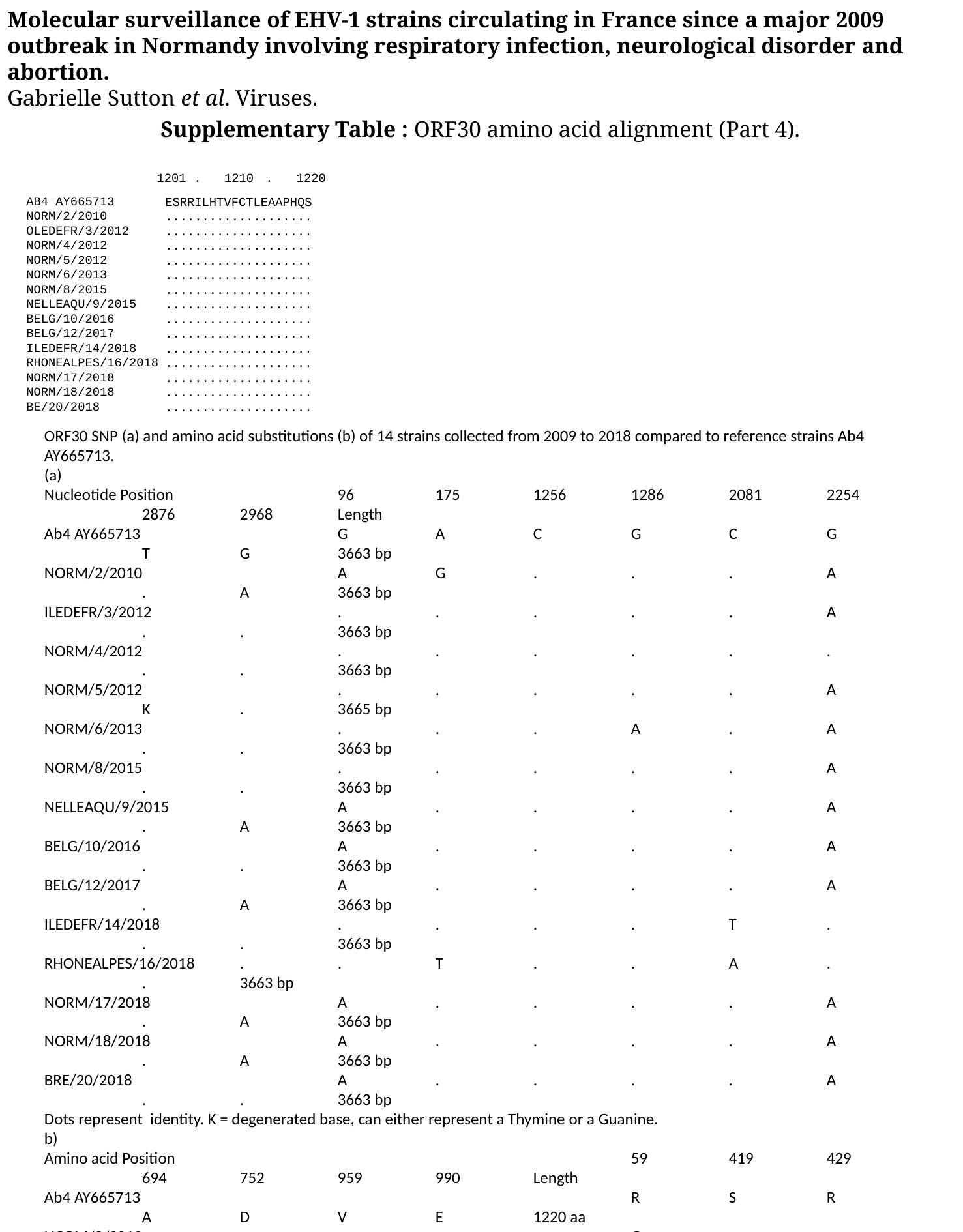

Molecular surveillance of EHV-1 strains circulating in France since a major 2009 outbreak in Normandy involving respiratory infection, neurological disorder and abortion.
Gabrielle Sutton et al. Viruses.
Supplementary Table : ORF30 amino acid alignment (Part 4).
1201
.
1210
.
1220
AB4 AY665713
NORM/2/2010
OLEDEFR/3/2012
NORM/4/2012
NORM/5/2012
NORM/6/2013
NORM/8/2015
NELLEAQU/9/2015
BELG/10/2016
BELG/12/2017
ILEDEFR/14/2018
RHONEALPES/16/2018
NORM/17/2018
NORM/18/2018
BE/20/2018
ESRRILHTVFCTLEAAPHQS
....................
....................
....................
....................
....................
....................
....................
....................
....................
....................
....................
....................
....................
....................
ORF30 SNP (a) and amino acid substitutions (b) of 14 strains collected from 2009 to 2018 compared to reference strains Ab4 AY665713.
(a)
Nucleotide Position		96	175	1256	1286	2081	2254	2876	2968	Length
Ab4 AY665713			G	A	C	G	C	G	T	G	3663 bp
NORM/2/2010		A	G	.	.	.	A	.	A	3663 bp
ILEDEFR/3/2012		.	.	.	.	.	A	.	.	3663 bp
NORM/4/2012		.	.	.	.	.	.	.	.	3663 bp
NORM/5/2012		.	.	.	.	.	A	K	.	3665 bp
NORM/6/2013		.	.	.	A	.	A	.	.	3663 bp
NORM/8/2015		.	.	.	.	.	A	.	.	3663 bp
NELLEAQU/9/2015		A	.	.	.	.	A	.	A	3663 bp
BELG/10/2016			A	.	.	.	.	A	.	.	3663 bp
BELG/12/2017			A	.	.	.	.	A	.	A	3663 bp
ILEDEFR/14/2018		.	.	.	.	T	.	.	.	3663 bp
RHONEALPES/16/2018	.	.	T	.	.	A	.	.	3663 bp
NORM/17/2018		A	.	.	.	.	A	.	A	3663 bp
NORM/18/2018		A	.	.	.	.	A	.	A	3663 bp
BRE/20/2018			A	.	.	.	.	A	.	.	3663 bp
Dots represent identity. K = degenerated base, can either represent a Thymine or a Guanine.
b)
Amino acid Position					59	419	429	694	752	959	990	Length
Ab4 AY665713						R	S	R	A	D	V	E	1220 aa
NORM/2/2010					G	.	.	.	N	.	K	1220 aa
ILEDEFR/3/2012					.	.	.	.	N	.	.	1220 aa
NORM/4/2012					.	.	.	.	.	.	.	1220 aa
NORM/5/2012					.	.	.	.	N	X	.	1220 aa
NORM/6/2013					.	.	K	.	N	.	.	1220 aa
NORM/8/2015					.	.	.	.	N	.	.	1220 aa
NELLEAQU/9/2015					.	.	.	.	N	.	K	1220 aa
BELG/10/2016						.	.	.	.	N	.	.	1220 aa
BELG/12/2017						.	.	.	.	N	.	K	1220 aa
ILEDEFR/14/2018					.	.	.	V	.	.	.	1220 aa
RHONEALPES/16/2018				.	L	.	.	N	.	.	1220 aa
NORM/17/2018					.	.	.	.	N	.	K	1220 aa
NORM/18/2018					.	.	.	.	N	.	K	1220 aa
BRE/20/2018						.	.	.	.	N	.	.	1220 aa
HSV Polymerase domain correspondance 	A	B	B 	C	C	D	D
Dots represent identity. X = unidentified amino acid (due to a degenerate nucleotide, see Table 6a)
A= pre-NH2 terminal domain B= 3’-5’ exonuclease domain C= palm domain D= thumb domain
